# Giant anomalous exciton-multiphoton nonlinearities in layered hybrid perovskites

**DOI:** 10.1038/s41377-026-02286-6

**Published:** 2026-08-25

**Authors:** Yanming Xu, Yi Liu, Chao Yu, Jinlong Xu, Wenqi Huang, Weichu Mo, Canhua Xu, Zhihua Sun, Yantang Huang, Xun Cao, Zhenda Xie, Ruifeng Lu

**Affiliations:** 1 Fujian Key Laboratory of Quantum Information and Quantum Optics, College of Physics and Information Engineering, Fuzhou University, Fuzhou, 350108 China; 2 National Laboratory of Solid State Microstructures, School of Electronic Science and Engineering, Nanjing University, Nanjing, 210093 China; 3 State Key Laboratory of Functional Crystals and Devices, Fujian Institute of Research on the Structure of Matter, Chinese Academy of Sciences, Fuzhou, 350002 China; 4 Institute of Ultrafast Optical Physics, Department of Applied Physics, Nanjing University of Science and Technology, Nanjing, 210094 China

**Keywords:** Nonlinear optics, Nonlinear optics

## Abstract

Directly full-color frequency-upconversion under high-order multiphoton absorption (MPA) nonlinearities in NIR-III (1550–1870 nm) and NIR-IV (2100–2300 nm) windows is highly desirable for advanced photonics and bioimaging. However, it has remained beyond the reach of current materials due to their inherently weak high-order MPA. Here, we demonstrate that, driven by Bloch oscillations of excitonic dipoles after nonlinear polarization, giant multiphoton nonlinearities can be achieved in layered hybrid perovskites to enable full-color upconversion in NIR-III/IV windows. The MPA cross-section values of the designed three Ruddlesden-Popper perovskites are found to be several orders of magnitude larger than those of the reported MPA materials. These advancements facilitate versatile applications previously inaccessible in NIR-III/IV windows, including full-color upconversion displays and multicolor information encryption. Our work highlights the prospect of efficient high-order MPA and opens avenues for investigating excitonic quantum dynamics in high-order nonlinear optics.

## Introduction

Frequency upconversion of near-infrared (NIR) photons into the visible (VIS) spectral region enabled by multiphoton absorption (MPA) provides a high-resolution approach for dynamic information resolution and optical imaging. The NIR region is divided into four main windows with high transparency in water and biological tissues: 700–1000 nm (NIR-I), 1000–1350 nm (NIR-II or NIR-IIa), 1550–1870 nm (NIR-III or NIR-IIb and IIc), and 2100–2300 nm (NIR-IV or NIR-IId)^1–5^. Among these, NIR-III/IV windows hold great promise that NIR-III, capable of the maximum tissue permeability is identified as the “golden window”^1^, and NIR-IV possesses the largest suppression in background Rayleigh scattering^2^. Recent studies on two-photon absorption (2PA) and three-photon absorption (3PA) under NIR-I/II excitation have made significant advancement in frontier photonic applications, such as high-resolution three-dimensional (3D) bioimaging^6–8^, 3D nanofabrication^9–11^, direct-writing of high-density photonic crystal^12,13^, manufacturing of integrated photonics device^14,15^, and strong-field photodetection^16^. These breakthroughs encourage further exploration into four- to six-photon absorption (4PA-6PA) in NIR-III/IV windows, aiming to enhance the performance of these applications. However, because 4PA, 5PA and 6PA belong to 7th, 9th and 11th perturbative nonlinearities, respectively, the weak high-order nonlinearities of existing MPA materials pose a formidable challenge. It thus underscores the need for new insights into the enhancement mechanisms of MPA nonlinearities.

The exciton, a quantized Coulomb-bound electron-hole pair that behaves as a boson, has been characterized as a dipole moment for nonlinear polarization^17–22^. Wannier excitons have been found to remain stable at room temperature in low-dimensional semiconductors with quantum confinement, making it possible to greatly enhance second- and third-order nonlinearities, including second harmonic generation^23–25^, four-wave mixing^26^, 2PA^27–29^, and nonlinear refractive index^30^. However, during the past decade, the underlying mechanisms, particularly the light-driving dynamics of excitonic dipoles and its contribution to higher-order nonlinearities (such as high-order MPA), are not yet fully understood despite numerous investigations, which are hindered by two primary challenges: (1) Distinguishing the features of nonlinear effects induced by transient excitonic dipoles from those originated from intrinsic atomic/molecular dipoles. (2) Numerous excitonic dipoles are required for achieving significantly enhanced high-order nonlinearity, necessitating materials with strong quantum and dielectric confinements^31^. Recent research has focused on Ruddlesden-Popper perovskites (RPPs), which are formed by interlayers of inorganic frameworks and organic cations. Benefiting from their programmable nanostructured multiple quantum-well (MQW) superlattices, the excitons in RPPs retain intralayer-confined Wannier-type properties^32,33^, presenting unexplored opportunity for investigating the contribution of excitonic behavior to high-order nonlinearities.

In this study, we demonstrated that strategically tailoring of both the structural components and MQW layer thickness in three RPPs can yield giant multiphoton nonlinearities towards NIR-III/IV windows, resulting in red-green-blue (RGB) upconversion emission in RPP bulks and full-color upconversion in nanoscale composites. Our findings reveal that these unprecedented nonlinearities originate from Bloch oscillations of excitonic dipoles (BOED) within the MQW structures, as evidenced by experimental observations of anomalous MPA evolutions (including a transition from isotropy to anisotropy with increasing pump intensity, and stronger polarization with reducing pump photon energy) and corresponding theoretical simulations. This work opens new avenues for exploring high-order nonlinear optical materials and advancing its frontier applications of MPA in NIR-III/IV windows.

## Results

### RPP design strategy and characterization

MPA characterizes a perturbative nonlinear polarization response to excitation intensity, where a *n*-photon absorption process is governed by the imaginary part of the (2*n*-1)-th order nonlinear susceptibility ${\chi }^{\left(2n-1\right)}(n\,=2,\,3,\,4\ldots )$. Accordingly, the third-order (${\chi }^{(3)}$), fifth-order (${\chi }^{(5)}$), and seventh-order (${\chi }^{(7)}$) nonlinear susceptibilities are responsible for 2PA, 3PA, and 4PA processes, respectively, and so forth^34^. Thus, to realize high-efficiency MPA nonlinearities under moderate laser intensities to avoid phototoxicity and material damage, a crucial approach is to enhance the imaginary part of high-order ${\chi }^{(n)}$. In the context of MPA processes, an approximation of ${\chi }^{(n)}$ can be simplified as ${\chi }^{\left(n\right)} \sim {\mu }^{n+1}/{{(\hslash \omega }_{0})}^{n} \sim {\left[{P}^{(1)}\right]}^{n+1}$, where μ is the dipole moment, ${\omega }_{0}$ is the applied field frequency, and ${P}^{(1)}$ is the linear polarization^34^. Therefore, a larger dipole moment inherently leads to increased ${P}^{(1)}$, thereby enhancing multiphoton nonlinearities.

Further considering the contribution of excitonic dipoles to optical nonlinearity^17–30^, designing RPPs capable of high-order multiphoton upconversion should prioritize the following aspects: (1) Utilizing strongly electronegative halide anions to induce a large bandgap in VIS region and facilitate high-order MPA. (2) Employing thin quantum well (i.e., few-layered inorganic sheets) with strong quantum confinement to enhance exciton binding energy and prevent exciton dissociation under intense excitation. (3) Maximizing the contrast of dielectric constants between the inorganic sheets and organic cations to reduce charge screening, thereby enhancing Coulomb attraction and enlarging exciton binding energy^35^. (4) Establishing strong interlayer hydrogen bonds and well-matched lattices at the abrupt organic-inorganic interface for ensuring high environment stability and robust resistance to damage under intense excitation.

**Fig. 1:**
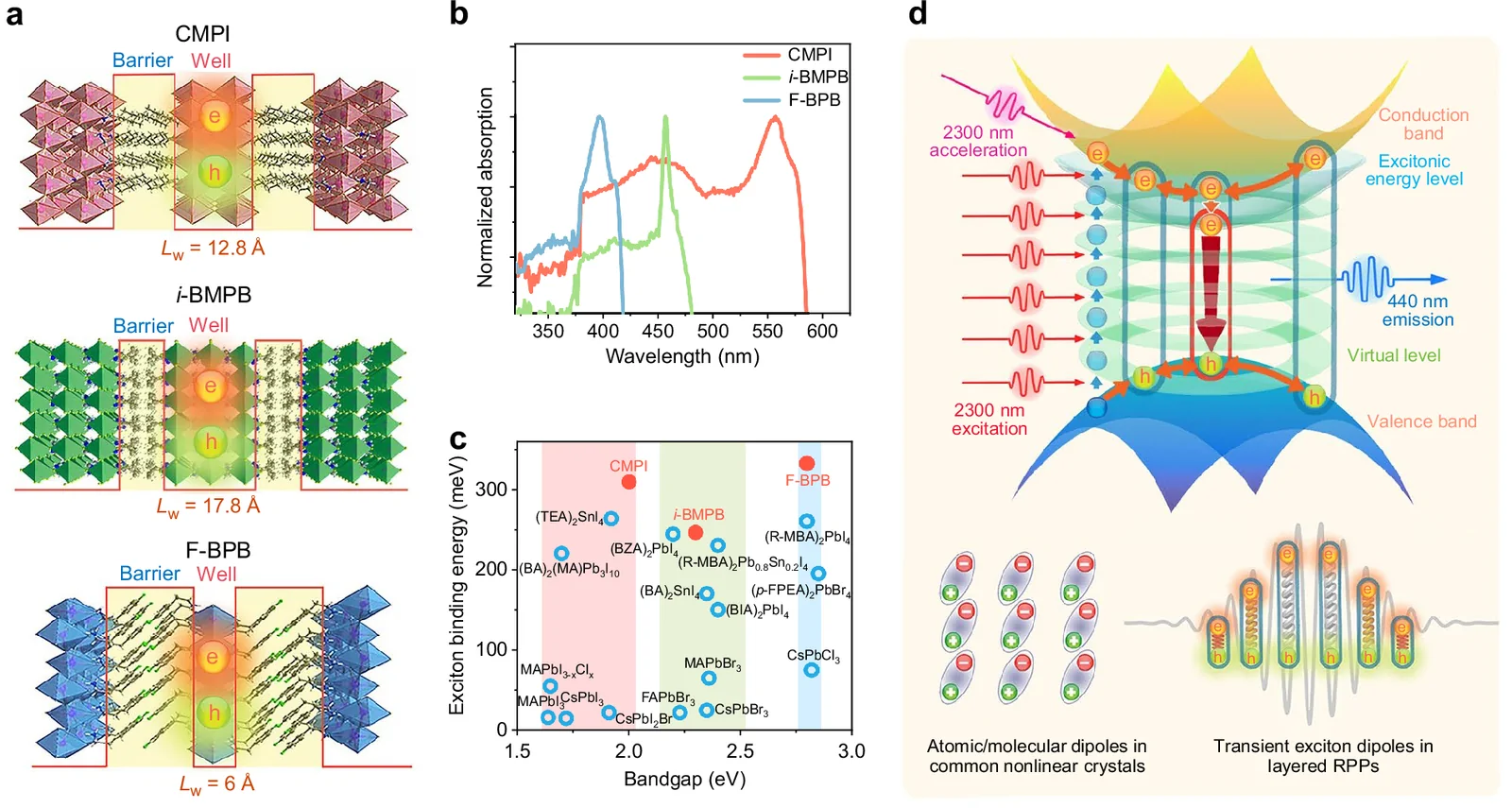
Structural properties and the principle of MPA upconversion for layered CMPI, *i*-BMPB, and F-BPB. **a** Diagram of the MQW nanostructures for top view in *ab* plane with alternative arrangements of inorganic perovskite sheets (well) and organic cation layers (barrier). **b** Linear optical absorption spectra with strong excitonic absorption peaks. **c** Comparison of the exciton binding energies (309 meV for CMPI, 246 meV for *i*-BMPB, and 332 meV for F-BPB) with typical perovskites with RGB gamut bandgaps. References: MAPbI_3_^36^, MAPbBr_3_^37^, MAPbI_3-x_Cl_x_^38^, FAPbBr_3_^39^, CsPbI_3_^40^, CsPbBr_3_^41^, CsPbI_2_Br^40^, CsPbCl_3_^42^, (BA)_2_(MA)Pb_3_I_10_^43^, (BIA)_2_PbI_4_^44^, (BZA)_2_PbI_4_^44^, (R-MBA)_2_PbI_4_^45^, (*p*-FPEA)_2_PbBr_4_^46^, (BA)_2_SnI_4_^47^, (TEA)_2_SnI_4_^48^, and (R-MBA)_2_Pb_0.8_Sn_0.2_I_4_^45^. **d** Top panel: microscopic mechanism for the enhancement of MPA nonlinearities enabled by dynamic BOED in the case of 6PA process. Bottom panel: comparison of the physical models between atomic/molecular dipoles and excitonic dipoles

Based on all the concerns mentioned above, we have designed three layered RPP structures: (CA)_2_(MA)Pb_2_I_7_ (CMPI, orthorhombic), (*i*-BA)_2_(MA)_2_Pb_3_Br_10_ (*i*-BMPB, orthorhombic), and (F-BEA)_2_PbBr_4_ (F-BPB, monoclinic), as illustrated in Fig. 1a (please see Supplementary Note 1 for detailed crystal structure characterizations). These three crystal structures feature inorganic frameworks composed of corner-sharing PbX_6_ (X = Br, I) octahedra that link two layers of hydrophobic organic spacer cations (CA, *i*-BA or F-BEA) along the *a*-axis through strong N-H ∙ ∙ ∙ X hydrogen bonds. All the structures exhibit inverse symmetry in *bc* plane, with about 45^o^ between the Pb-X bonding and in-plane *b* (or *c*)-axis directions (the left panels of Fig. S2a–c). Such an interlayer arrangement constitutes wells and barriers, forming a two-dimensional (2D) superlattice motif that enables a platform for tailoring individual bandgaps within the RGB gamut range (the middle panels of Fig. S2a-c). Further, theoretical analyses on the partial density of states and band alignments certify the crucial role of inorganic layers in establishing the natural Type-I QWs of these three RPPs (the right panels of Fig. S2a-c). Moreover, owing to the high dielectric contrast between the well and barrier layers, the confinement potential energies of these three structures are significantly higher than those of many reported RPPs with bandgaps in RGB gamut range (Figs. S2 and S3; detailed data are listed in Table S2), which is advantageous for stabilizing excitonic behaviors.

After synthesizing bulk single crystals of the three RPPs (see Materials and methods for details), their linear optical absorption spectra were measured as shown in Fig. 1b. Figure 1b manifests distinct absorption peaks at 556 (CMPI), 462 (*i*-BMPB) and 397 nm (F-BPB), corresponding to the lowest energy of excitonic transition^35^. Their linear photoluminescence (PL) spectra are centered at 640, 530, and 442 nm, respectively (Fig. S4), well located at the RGB gamut. From these measurements, the optical bandgap energies are estimated to be approximately 1.8 (CMPI), 2.2 (*i*-BMPB), and 2.8 eV (F-BPB). As shown in Fig. 1c, the measured exciton binding energies *E*_b_ are 309 meV for CMPI, 246 meV for *i*-BMPB, and 332 meV for F-BPB (see Supplementary Note 3 for detailed measurement). These values are substantially larger than those reported for prominent 2D/3D perovskites with RGB gamut bandgaps^36–48^ (detailed data are listed in Table S3), indicating their great potential in excitonic multiphoton nonlinearity.

### Origin of giant multiphoton nonlinearities

Before delving into the experimental investigation on the MPA properties of these RPPs, it is essential to discuss the physical origins of the possible strong multiphoton nonlinearities in a robust excitonic system. According to classic theories regarding resonant optical nonlinearities of excitonic dipoles in systems with strong dielectric or quantum confinement^17–19^, we anticipate that the microscopic MPA response of the as-grown RPPs is linked to the coherent dynamics of excitonic dipoles when subjected to femtosecond (fs) laser driving.

To elucidate the physical origin of giant multiphoton nonlinearities, the high-order MPA (6PA) induced interband transitions and upconversion PL process are taken as an example. As schematically shown in Fig. 1d, in the initial state of laser pulse excitation, some valence electrons in the well are excited from the valence to conduction band through virtual intermediate levels via atomic/molecular dipole polarization in crystals. Simultaneously, exciton states are formed with tight Coulomb attraction between the excited electrons and created valence holes. This band-to-band correlation can induce significant polarization of the excitonic dipoles. Laser pulses then drive these excitons moving within valence and conduction bands.

In relatively weak excitonic systems, such as 3D perovskites with low exciton binding energies, strong excitation can drive excitons to higher energy levels, causing them to dissociate into free electrons and holes. In the absence of significant scattering, these electrons and holes can be accelerated to move periodically within their respective bands and eventually diffract at the Brillouin zone boundaries. This delocalized dynamic is periodical at Bloch frequency and known as intraband Bloch oscillations of electrons (holes)^49–51^, producing nonlinear intraband currents that are widely recognized as the dominate source of high-harmonic generation in solids^49–53^.

However, it is important to note that the designed RPPs here exhibit strong excitonic characteristics with large exciton binding energies, making excitons dissociation difficult even under strong laser excitation. In this regime, the electron and hole remain bound and move together as a unit following the laser-field driving. Consequently, the Bloch oscillations become localized in the vicinity of conduction band minima (CBM) and valence band maxima (VBM), governed by the combined effect of the laser-field driving and excitonic Coulomb binding. Here, we highlight this local motion of excitons as BOED, in which the coherent superposition of excitonic dipoles results in a new source of collective linear polarization moment ${P}^{(1)}$. The component of the electric dipole moment parallel to the electric field vector (i.e., the polarization direction) of pump laser can contribute an enhancement of each-order nonlinear susceptibility for MPA, amplifying electron transition, producing a high density of excitonic dipoles, and eventually leading to giant multiphoton nonlinearities. Exciton recombination occurs through decoherence from the Bloch states near the CBM and VBM, followed by relaxation of electrons from the conduction band to the excitonic energy level and subsequently back to the valence band. This process is accompanied by the emission of a cascade of upconversion photons.

It is noteworthy that in conventional nonlinear optics for a crystal system, as depicted in the bottom figure of Fig. 1d, the generated atomic/molecular dipoles exhibit similar polarization strength due to the consistent optical and crystal field regulations. However, in the BOED scenario, excitonic dipoles experience different polarization strengths because they occupy various energy states during the oscillations along the bands. This distinction suggests that nonlinearities governed by excitonic dipole moments should be more pronounced at higher-order MPA, overcoming the limitations of conventional nonlinearities. This scenario is further supported by the stronger enhancement effect of upconverted PL at higher-order MPA, as discussed in the subsequent section.

### Measurement of giant multiphoton nonlinearities in RPP bulks

**Fig. 2:**
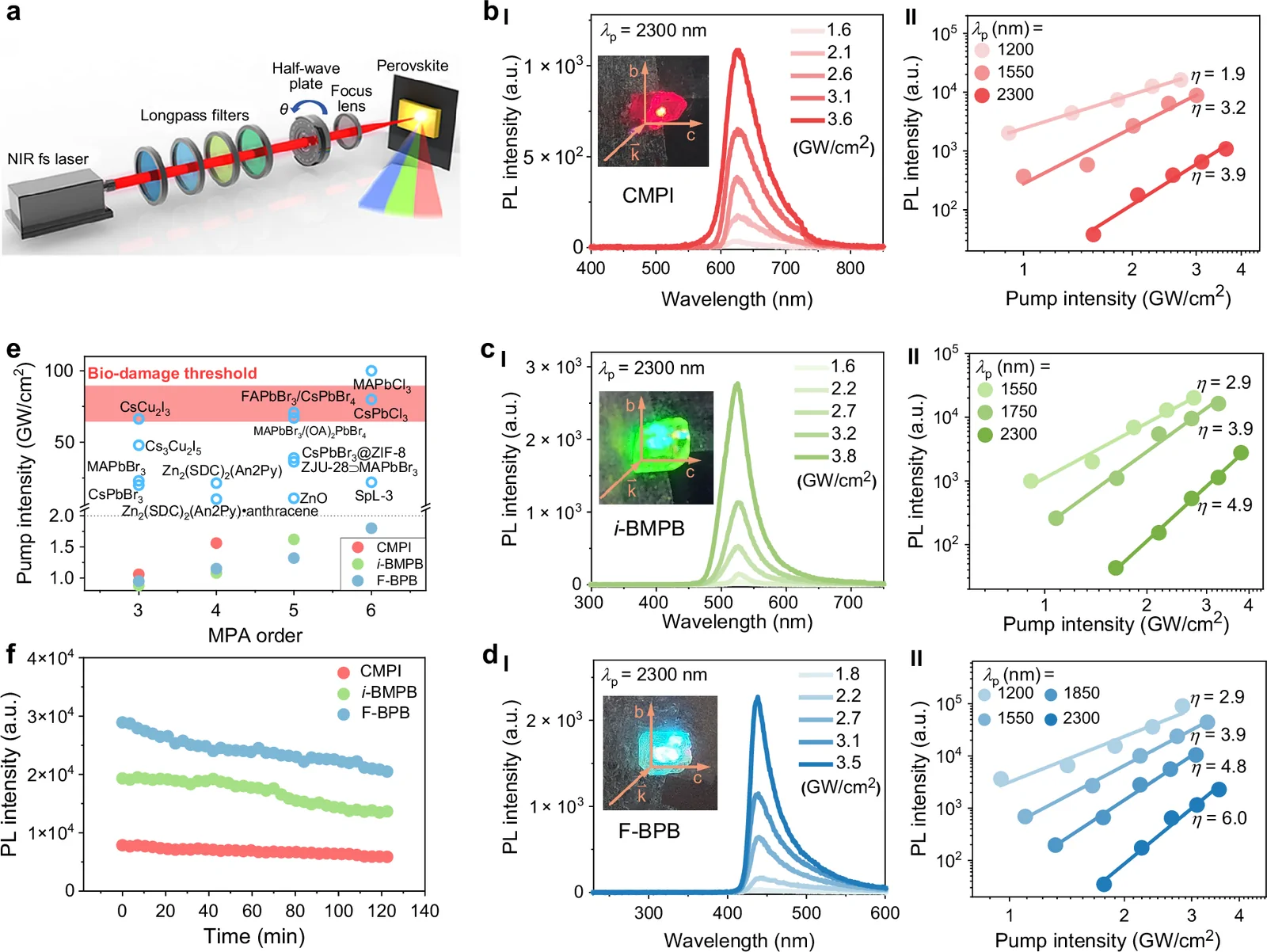
MPA-induced RGB upconversion emission from the bulks of CMPI, *i*-BMPB and F-BPB. **a** Schematic diagram of the experimental setup. **b–d** (I) PL spectra under different pump intensities (*λ*_p_ = 2300 nm). Inset: photographs of upconversion PL from individual RPP bulks under NIR-IV pumping (*λ*_p_ = 2300 nm). Orange arrows indicate the wave vector (***k***) and the crystallographic *b*- and *c*-axes. (II) Logarithmic plot of the MPA PL peak strength dependence on pump intensity at different wavelengths within NIR-II/III/IV windows. The solid lines represent fitting with the power-law equation ${I}_{{\rm{p}}}^{\eta }$, where the extracted values of η follow an $\eta \approx n$ dependence for n-photon absorption. This verifies a perturbative scaling of nonlinear optics for MPA process. **e** Comparison of the minimum pump intensity in 3PA-6PA processes with representative results reported from other materials. The pump intensity ${I}_{\min }$ refers to the required minimum pump intensity to excite the MPA-induced upconversion PL. References: MAPbBr_3_^55^, CsPbBr_3_^55^, MAPbBr_3_/(OA)_2_PbBr_4_^55^, Zn_2_(SDC)_2_(An2Py)^56^, Zn_2_(SDC)_2_(An2Py)•anthracene^56^, CsPbCl_3_^57^, Cs_3_Cu_2_I_5_^58^, CsCu_2_I_3_^58^, ZnO^59^, ZJU-28⊃MAPbBr_3_^60^, CsPbBr_3_@ZIF-8^61^, FAPbBr_3_/CsPbBr_3_^62^, SpL-3^63^, and MAPbCl_3_^64^. The red horizontal bar indicates the bio-damage threshold referring to ref. ^65–67^. **f** PL intensity variation with time under long-time fs laser irradiation (*λ*_p_ = 1200 nm and *I*_p_ = 7.2 GW/cm^2^). The duration is 120 min with an interval of 3.5 min

The experimental setup for characterizing multiphoton nonlinearities of the three as-grown bulk RPPs is presented in Fig. 2a. Stable MPA upconversion PL was observed with broadband tuning of pump wavelength (*λ*_p_) from NIR-I to NIR-IV windows (800–2300 nm). The corresponding PL spectra and photographs are shown in Fig. 2b(I)-d(I) (*λ*_p_ = 2300 nm) and Fig. S6 (other pump wavelengths). The peaks of the PL spectra for CMPI, *i*-BMPB, and F-BPB are centered at 625, 525, and 440 nm, respectively, well within the RGB gamut. Further, the dependence of PL intensity on pump intensity (*I*_p_) was studied under NIR-II/III/IV excitation (Fig. 2b(II)–d(II)). The power-law fitting, denoted by exponential function ${I}_{{\rm{p}}}^{\eta }$ ($\eta \approx n$ for n-photon absorption), proves that the PL-pump dependence of CMPI, *i*-BMPB, and F-BPB fulfills the perturbative nonlinear-optics characteristics of 2PA-4PA, 3PA-5PA, and 3PA-6PA processes, respectively^54–57^. Notably, the minimum pump intensities (${I}_{\min }$) for 3PA-6PA are significantly lower than those of reported materials^55–64^ and well below the bio-damage threshold^65–67^, as demonstrated in Fig. 2e (detailed data are listed in Table S4), highlighting their potential for biophotonics applications.

To accurately investigate the high-order nonlinearities related to MPA, open aperture (OA) Z-scan experiments were performed to determine the absorption coefficients and absorption cross-sections for MPA processes in the three RPP bulks (see details in Supplementary Note 5). As anticipated, the results for 3PA-6PA (Table 1) surpass those of almost all the reported MPA materials to date (detailed comparison is provided in Table S5). The absorption cross-sections for 5PA (9.1 × 10^−134^ cm^10^s^4^photon^−4^ for F-BPB) and 6PA (4.2 × 10^−163^ cm^12^s^5^photon^−5^ for F-BPB) are approximately three and six orders of magnitude larger than those for reported MPA materials, respectively^55,59,63^.

**Table 1:** Multiphoton absorption cross-section *σ*_n_ in NIR-II/III/IV windows

Material type	3PA *σ*_3_ (cm^6^s^2^photon^−2^)	4PA *σ*_4_ (cm^8^s^3^photon^−3^)	5PA *σ*_5_ (cm^10^s^4^photon^−4^)	6PA *σ*_6_ (cm^12^s^5^photon^−5^)
CMPI Bulk crystals Nano crystals	(NIR-III, 1550 nm) 2.0 × 10^−76^ 1.7 × 10^−72^	(NIR-IV, 2300 nm) 1.3 × 10^−105^ 8.5 × 10^−102^	-	-
*i*-BMPB Bulk crystals Nano crystals	(NIR-II, 1200 nm) 5.1 × 10^−75^ 6.3 × 10^−72^	(NIR-III, 1750 nm) 1.8 × 10^−104^ 6.2 × 10^−102^	(NIR-IV, 2300 nm) 4.5 × 10^−133^ 3.9 × 10^−131^	-
F-BPB Bulk crystals Nano crystals	(NIR-II, 1200 nm) 2.6 × 10^−76^ 5.3 × 10^−72^	(NIR-III, 1550 nm) 7.8 × 10^−105^ 5.8 × 10^−102^	(NIR-III, 1850 nm) 9.1 × 10^−134^ 2.0 × 10^−131^	(NIR-IV, 2300 nm) 4.2 × 10^−163^ 9.5 × 10^−161^

Furthermore, we monitored the MPA upconversion PL intensities of the three RPP bulks under long-time (120 min) intense pumping (*λ*_p_ = 1200 nm, *I*_p_ = 7.2 GW/cm^2^) to evaluate the MPA stability (see details in Fig. S7). As depicted in Fig. 2f, the PL intensities remain ~75% (CMPI), ~71% (*i*-BMPB), and ~72% (F-BPB) of the initial values, indicating superior laser-damage resistance. Additionally, these RPPs exhibit excellent long-term environmental stability, retaining over 90% of their initial PL intensity after storage in a vacuum box for 15 months (Fig. S8). These giant multiphoton nonlinearities are attributed to the BOED dynamics, supported by the experimental observations of anomalous MPA evolutions in anisotropy strength as discussed in the following.

### Anomalous anisotropy of MPA upconversion PL

The exceptional single-crystalline nature of the three RPP bulks allows us to further investigate the PL polarization dependence. The anisotropic evolutions of MPA upconversion PL under different crystallographic orientation in *bc* plane were measured by employing a half-wave plate to rotate the angle *θ* between the *c*-axis direction of the samples and the linear-polarized direction of the pump laser (Fig. 2a). The results reveal significant difference between one-photon absorption (1PA) and MPA processes. The 1PA PL remains isotropic regardless of pump strength (see details in Fig. S14), but the MPA upconversion PL exhibits exotic anisotropic behavior that depends on the pump strength.

**Fig. 3:**
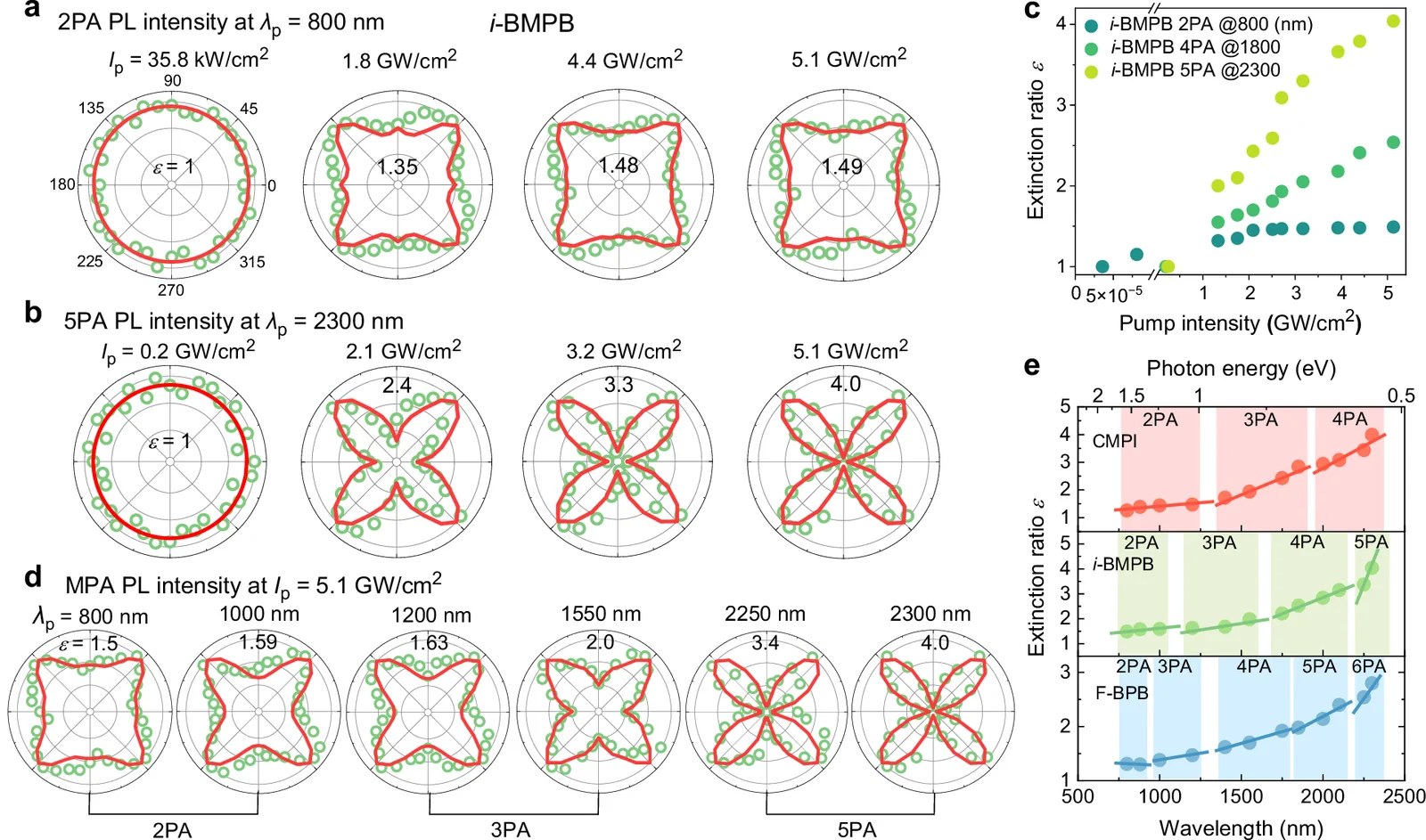
Anomalous evolutions of MPA anisotropy in the bulk RPPs. **a** Representative polar plots demonstrate the orientation evolutions of 2PA PL (*λ*_p_ = 800 nm) in *i*-BMPB for large-scale variation of pump intensity. The minima at 0^o^ and 180^o^ along *c*-axis, 90^o^ and 270^o^ along *b*-axis; while the maxima at 45^o^, 135^o^, 225^o^, and 315^o^ corresponds to the PbX_6_ octahedra corner-connecting directions. **b** Orientation evolutions of 5PA PL (*λ*_p_ = 2300 nm) in *i*-BMPB for available scale of pump intensity. **c** Retrieved extinction ratios as a function of pump intensity in different MPA processes for *i*-BMPB. **d** Orientation evolutions of MPA PL in *i*-BMPB at different pump wavelengths under a constant pump intensity of 5.1 GW/cm^2^. The green hollow dots in (**a**), (**b**), and (**d**) are experimental data, while the red lines depict the simulation results based on BOED. **e** Extinction ratios as a function of pump wavelength under a constant pump intensity of 5.1 GW/cm^2^ for the three RPPs. The solid lines are linear fittings for the experimental data in each order of MPA processes

To illustrate this anisotropic evolution in MPA upconversion PL, we used *i*-BMPB as an example and employed both a Ti:sapphire oscillator and an amplifier to realize a wide tuning range of pump intensity at *λ*_p_ = 800 nm. The 2PA PL strength exhibits an anomalous evolution from isotropy (at *I*_p_ = 35.8 kW/cm^2^) to anisotropy with increasing pump strength. This anisotropy displays distinct in-plane four-fold symmetry with maxima at *θ* = 45°, 135°, 225°, and 315° (see the corresponding polar plots in Fig. 3a). Further increasing the pump strength leads to a gradual enhancement of anisotropy strength, with the extinction ratio *ε* (defined as the ratio of PL intensity between *θ* = 45^o^ and 0^o^) increasing from 1 to ~1.5 (Fig. 3a, c).

**Fig. 4:**
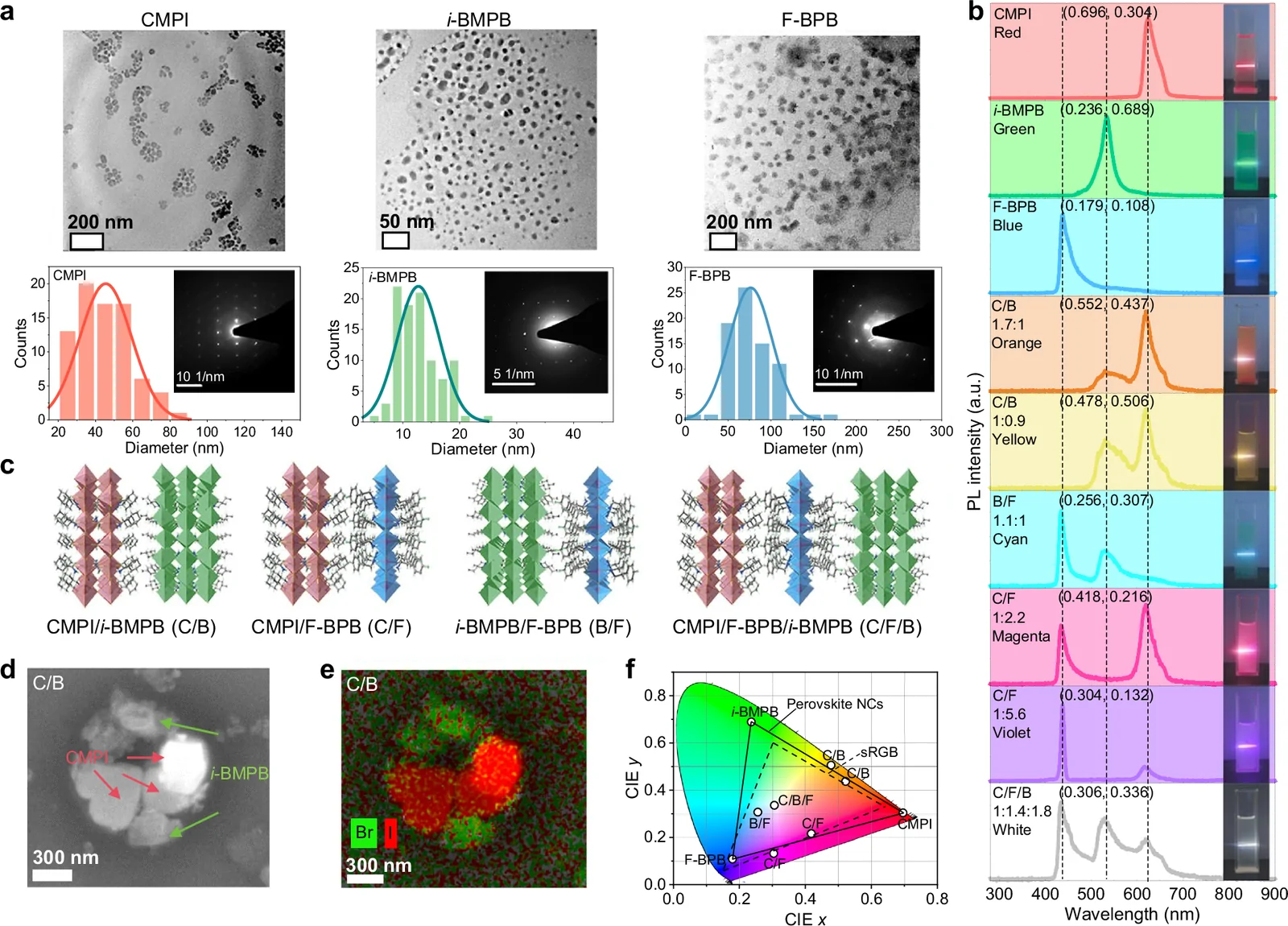
Full-color MPA upconversion PL emission from NCs of CMPI, *i*-BMPB, F-BPB and their composites. **a** Top panels: TEM images of CMPI, *i*-BMPB, and F-BPB NCs. Bottom panels: corresponding size distributions and SAED patterns. **b** Full-color MPA PL spectra and photographs including eight main VIS colors and white color pumped by NIR-III/IV lasers. The marked ratios denote the mixing molar ratios of the three NCs in each composite. The inset numbers in brackets refer to the CIE1931 coordinates. **c** Schematics for four types of binary/ternary NC composites. **d, e** SEM image and EDS elemental mapping of C/B composite. **f** CIE coordinates (circle) as marked in (**b**) plotted on a chromaticity diagram. The full-color MPA upconversion encompasses a broader range of color gamut (solid line) compared to that of sRGB (dotted line)

For higher-order MPA, similar behavior of growing anisotropy was recorded. Polar plots of 5PA (Fig. 3b) and 4PA (Fig. S16c) with corresponding *ε* values presented in Fig. 3c demonstrate this behavior. Furthermore, *ε* increases monotonically with the pump wavelength, even within the same MPA order (i.e., the same order of optical nonlinearity), as shown in Fig. 3d and the middle panel in Fig. 3e. The polarization-dependent absorbance exhibits the same anomalous anisotropic behavior as the PL (the corresponding polar plots are given in Fig. S16a–c). This indicates that the anisotropic PL evolutions result directly from the anisotropy of MPA process, without significant post-absorption energy-loss pathways such as defect-state-induced luminescence. Likewise, very similar anisotropy evolutions were experimentally observed in CMPI and F-BPB (the top and bottom panels of Fig. 3e and Fig. S19, see Figs. S17 and S18 for detailed polar plots).

These exotic anisotropy evolutions of MPA upconversion PL cannot be simply interpreted by conventional nonlinear optics based on atomic/molecular dipole moments^34^, as discussed below. (1) The symmetry of atomic/molecular dipole polarization depends on the inversion symmetry of crystal structure, which means that the integrated nonlinear susceptibility in each crystallographic direction remains constant under laser excitation. In this regard, the PL symmetry after MPA should not change with pump intensity at a fixed pump wavelength, thus should not induce transition from isotropy to anisotropy. (2) Conventional laws in nonlinear optics also suggest that longer pump wavelengths (i.e., lower photon energies) result in weaker polarization of atomic/molecular dipoles, and thus a lower extinction ratio. This is contrary to our experimental observations, where an increased extinction ratio is observed with longer wavelengths, indicating a need for a new understanding of high-order nonlinear effects in our experiments.

To address the anisotropy of MPA upconversion PL within these RPPs, we explore the role of BOED in determining the symmetry of MPA. As previously discussed, BOED is localized within the energy bands, making the anisotropy of excitonic nonlinearities dependent upon the band structure. First-principles calculations (Fig. S20) reveal that the energy bands of the RPPs exhibit a clear four-fold symmetry from CBM and VBM (**Γ** point) to the Brillouin zone boundary with anisotropy dispersion. Also, we simulated the interaction between the laser field and excitonic dynamics in RPPs by solving two-band semiconductor Bloch equations (SBEs) integrated with excitonic dipoles and anisotropy dispersion of energy bands (see Materials and methods for details). Most of the excitons generated through MPA processes initially localize around **Γ** point because the energy difference between CBM and VBM is minimum and the corresponding interband transition probability is maximum. The excitons can acquire momentum from the laser field and then oscillate in ***k*** space, with the additional momentum determined by vector potential $A\left(t\right)=-{\int }_{0}^{t}E({t}^{{\prime} }){{dt}}^{{{\prime} }}$ of laser field.

Deduced from Eqs. 1 and 2 in Materials and methods, the maximum vector potential ${A}_{\max }\propto {E}_{0}{\lambda }_{{\rm{p}}}$, where ${E}_{0}$ is the amplitude of the laser electric field. For short pump wavelength (i.e., low-order MPA regime) and low pump intensity, the A(t) value is small, leading to motion and localization of the excitonic dipoles very close to **Γ** point. Given that the energy bands of the three RPPs are approximately isotropic within the region very close to **Γ** point (Fig. S20), the MPA dynamics behave as isotropic or weak anisotropic. With increasing the laser intensity or pump wavelength (i.e., higher-order MPA regime), A(t) becomes larger, driving the excitons into anisotropic ***k*** space to facilitate BOED tracing along the bands at *θ* = 45°, 135°, 225°, and 315°, thereby generating PL dynamics from MPA upconversion with strong anisotropy. Theoretical simulations of BOED along these anisotropic energy bands align with the experimentally observed anomalous anisotropic evolutions of MPA upconversion PL (results for *i*-BMPB are presented in Fig. 3a, b, d and Fig. S16c, results for CMPI and F-BPB are shown in Figs. S17 and S18).

### Full-color MPA upconversion in nanoscale RPPs

To take full advantage of the giant multiphoton nonlinearities enabling RGB upconversion, we explore full-color upconversion using nanostructured RPPs to meet the demands of microimaging applications. High-yield preparation of low-dimensional nanocrystals (NCs) was conveniently realized through ultrasonic exfoliation of the three RPP bulks (see Materials and methods for details). Transmission electron microscope (TEM) images reveal that the mean sizes of the as-prepared NCs of CMPI, *i*-BMPB, and F-BPB are 45, 13, and 70 nm, respectively (upper figures of Fig. 4a, with corresponding selected area electron diffraction (SAED) patterns presented in the bottom figures of Fig. 4a). It is important to emphasize that obtaining clear high-resolution TEM images for characterizing crystal structure in more micro scale is challenging due to the high resistance of the three RPPs induced by robust excitonic effects (approximately 10^9^ Ω·cm, measured by the space charge limited current method). Despite this, the intrinsic single-crystal structures of the RPPs are preserved in the NCs since they are merely physically exfoliated from the single-crystal bulks without any chemical re-treatment.

As anticipated, the three NC samples also demonstrated efficient high-order MPA upconversion under NIR-III/IV laser excitation (see Fig. 4b for the MPA PL spectra and photographs). The PL peaks exhibit a slightly blue shift within 5 nm with respect to their bulk counterparts due to the enhanced quantum confinement of low-dimensional nanostructures, yet they still lie within the standard RGB (sRGB) gamut. The stronger quantum confinement of the NCs also results in larger MPA cross-sections compared to the RPP bulks (Table 1). The 5PA and 6PA absorption cross-sections of F-BPB NCs are five and eight orders of magnitude larger, respectively, than those reported for other MPA materials^55,59,63^ (see detailed comparison in Fig. S13 and Table S5).

By mixing the RGB primary colors with appropriate ratios of the three NC samples, a wide range of additive colors can be achieved. As presented in Fig. 4b, we prepared several mixing suspensions based on the RGB intensity ratios in the CIE color model. These suspensions include CMPI/*i*-BMPB (C/B), CMPI/F-BPB (C/F), *i*-BMPB/F-BPB (B/F), and CMPI/F-BPB/*i*-BMPB (C/F/B), enabling MPA upconversion PL in five main colors (orange, yellow, cyan, magenta, and violet) and in white color under NIR-III/IV excitation. Further, as schematically illustrated in Fig. 4c, a series of binary and ternary composites were fabricated by depositing different mixing suspensions on a substrate, followed by rapid drying to form dense NC films.

Here we choose the C/B sample as a representative to investigate the properties of the composites. As depicted in Fig. 4d, e, the C/B composites exhibit well-formed structures as observed in scanning electron microscope (SEM) image and energy-dispersive X-ray spectroscopy (EDS) elemental mapping. It should be noted that, similar to the TEM measurement, the SEM resolution is also hindered by the high resistance of these RPPs. Thus, the SEM images in Fig. 4d, e were recorded by employing larger-size NC samples (prepared under shorter ultrasonic exfoliation time) than those in Fig. 4a. But it can be inferred that successful formation of nanocomposites with smaller NCs has been achieved considering the identical fabrication processes. The SEM and EDS images of C/F and B/F also reveal their composite morphology, indicating successful implementation of composite fabrication (Fig. S21). Furthermore, time-resolved photoluminescence (TRPL) measurements (Figs. S22 and S23) provide clear evidence of strong interactions within the composites. The large-bandgap NCs exhibit a shortened PL lifetime, while the small-bandgap NCs show a prolonged lifetime, indicating efficient resonance energy transfer. These results not only confirm the successful formation of nanoscale composites but also help to explain slight spectral blue-shift observed in Fig. 4b.

The MPA upconversion PL colors of these NC composites are in line with those of mixing suspensions, according to the calculations of CIE coordinates marked in Fig. 4b. Figure 4f depicts that the MPA upconversion covers an RGB triangle’s area with 7.7% more perceptible colors than sRGB region (the calculation method is given in Supplementary Note 8). Moreover, owing to the high structural stability of the three RPPs, the composites exhibit good PL stability and long-term damage resistance even under intense excitation (*I*_p_ = 9.2 GW/cm^2^) for 90 min (we take C/F as an example to demonstrate the stability measurement in Fig. S24). These exceptional performances suggest that these NC composites are promising candidates for full-color upconversion displays of NIR-III/IV at the nanoscale. Overall, these results provide strong evidence for the presence of BOED and determine its crucial role in enhancing MPA nonlinearities. We speculate that BOED could be a universal dynamic in robust excitonic systems, which may pave the way for developing material physics towards robust high-order optical nonlinearities.

### Applications of full-color MPA upconversion nonlinearities

**Fig. 5:**
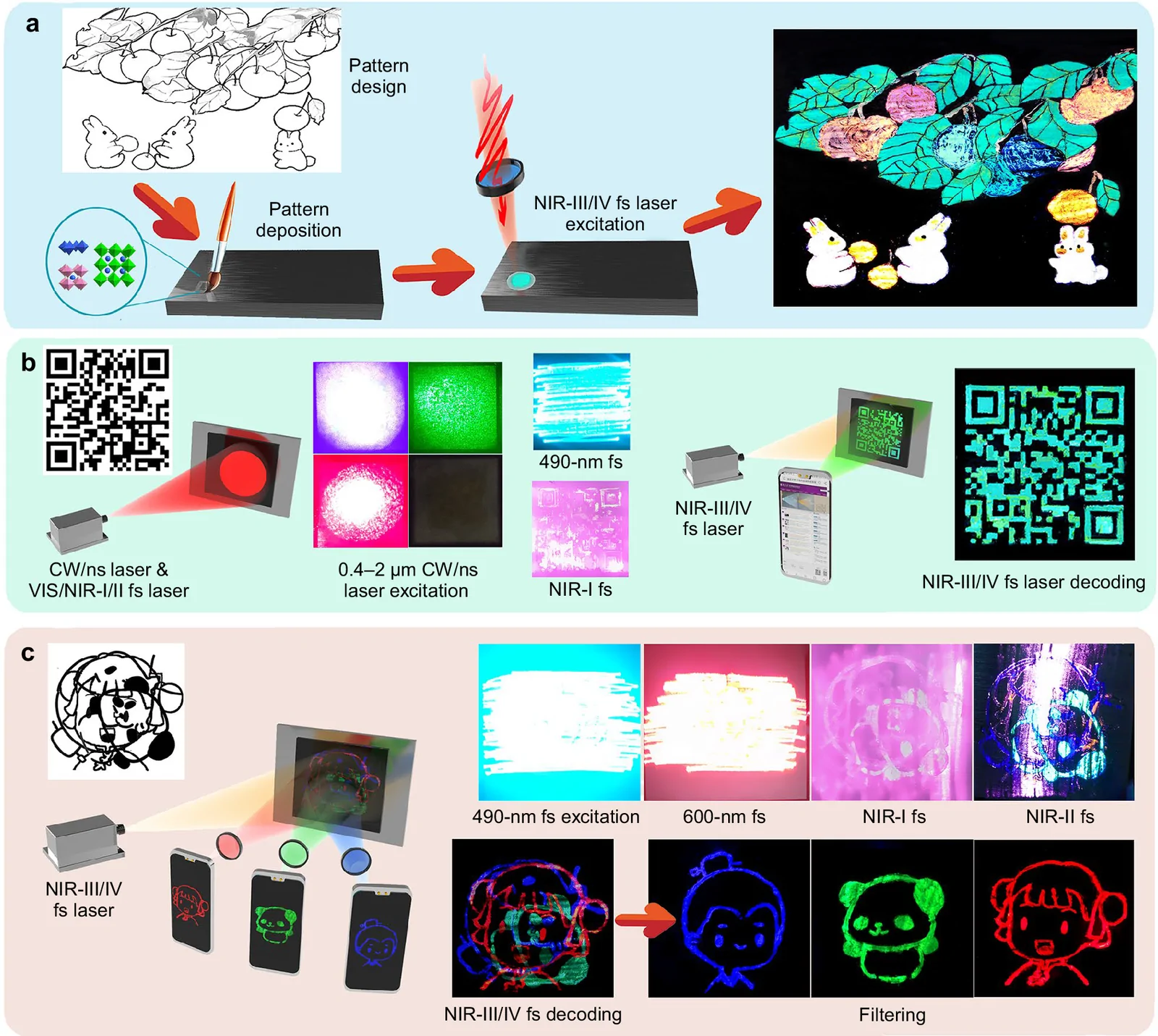
Paradigm of high-order MPA upconversion in full-color upconversion displays and multicolor information encryption in NIR-III/IV windows. **a** Implementation procedure of full-color upconversion displays prepared using single and composite NCs. **b** Concept of using NIR-III/IV fs laser to decode information. **c** Illustration of high-confidentiality multi-layer decryption by using multi-color MPA, wherein decoding information requires both NIR-III/IV pumping and spectrum filtering

Inspired by the robust full-color MPA upconversion, we explored its potential applications in upconversion displays. Initially, we deposited single NCs as well as binary and ternary NC composites onto a substrate to demonstrate a proof of concept for full-color upconversion displays with point-by-point scanning the substrate under NIR-III/IV laser pumping (pulse width ~120 fs, repetition rate 5 kHz). To the best of our knowledge, this is the first reported instance of full-color MPA upconversion displays in NIR-III/IV windows (Fig. 5a, see detailed implementation methods in Supplementary Note 9). This innovation may hold promise for advancing full-color NIR-III/IV bioimaging in non-invasive in vivo microscopy applications by using these NCs as fluorophores. Although the presence of Pb element in the RPPs poses a challenge for bioimaging applications, this issue can be addressed by encapsulating the NC with silica or biocompatible core-shell structures as certified by a lot of successful works^55,68–73^. In particular, the solvent-free encapsulation strategies are demonstrated to be suitable for the soluble RPPs, such as in-situ photopolymerization of silicones^68^ and vapor-phase deposition of dense silica^69^ or fluorinated silane layers^70^.

The giant high-order multiphoton nonlinearities also offer an intriguing opportunity for applications in information decoding based on the upconversion display strategy. As shown in Fig. 5b, utilizing NIR-III/IV fs lasers scanning to excite 4PA-6PA effects, a mobile phone equipped with a standard silicon CCD can readily identify a distinct quick-response (QR) code fabricated by *i*-BMPB NCs. In contrast, excitation with common VIS continuous-wave (CW) lasers results in weak PL, and background noise making code retrieval impossible. Even NIR nanosecond (ns) laser fails to excite high-order MPA PL, resulting in an all-black picture. Besides, VIS and NIR-I/II fs lasers often cause CCD saturation because they can easily induce efficient 1PA and 2PA effects of CCD.

The potential of colorful MPA for multi-layer information encryption based on various MPA upconversion is further demonstrated in Fig. 5c. Compared to common method with NIR-I/II light, this scheme based on NIR-III/IV MPA allows for clearer decoding and decryption with higher spatial resolution, profited from the better scattering suppression of longer wavelengths. This increased resolution enables higher information storage density and superior potential for super-resolution image encryption^74^. Moreover, this specificity offers enhanced information security compared to conventional methods that use common CW lasers.

## Discussion

In summary, we propose a solution for achieving full-color upconversion in NIR-III/IV windows based on the high-order multiphoton nonlinearities of RPPs. Our investigation reveals anomalous evolutions of MPA, including transition from isotropy to anisotropy in response to excitation intensity and enhanced anisotropy excited at longer wavelength. These effects, unexpected for traditional mechanisms from atomic/molecular dipoles, should be attributed to the instantaneous excitonic dipoles with Bloch oscillations. Our findings could advance the concepts and technologies of high-order nonlinear optics and ultrafast excitonic dynamics. In addition, the giant multiphoton nonlinearities are promising in various frontier applications, including NIR-III/IV bioimaging, high-confidentiality information security, high-capacity 3D optical data storage, 3D microfabrication for integrated photonics devices, broadband optical power limiting, and laser direct-writing for nanoscale photonic crystals.

## Materials and methods

### Chemical reagents

Cyclohexanemethylamine (97%, Aladdin), isobutylamine (99%, Aladdin), 4-fluorophenethylamine (98%, Aladdin), methylamine (40% in H_2_O), lead acetate trihydrate (Pb(Ac)_2_, 99.5%, Aladdin), hydrobromic acid (HBr, 48%, Aladdin), hydroiodic acid (HI, 48%, Aladdin). All the chemical reagents were bought and used without further purification.

### Synthesis of bulk crystals

For CMPI and *i*-BMPB, cyclohexanemethylamine/isobutylamine, methylamine, and Pb(Ac)_2_ were stoichiometrically dissolved in the solutions of hydrobromic/hydroiodic acid; while for F-BPB, stoichiometric amounts of 4-fluorophenethylamine and Pb(Ac)_2_ were dissolved in the concentrated solution of hydrobromic acid. Single-crystal RPPs were finally obtained through the temperature cooling method.

### Preparation of NC solution

The individual NC solution of CMPI, *i*-BMPB, and F-BPB was prepared by directly stripping the crystal flakes through high power (~960 W) ultrasonication for ~600 min and magnetic stirring for ~12 h, followed by centrifuged at 8000 rpm for ~30 min, finally the supernatant was collected. Dichloromethane (DCM) was chosen as the solvent.

### Crystal characterization

The linear absorption spectra were measured at room temperature on the Perkin-Elmer Lambda 900 UV-visible-NIR spectrometer. TEM images and SAED patterns were obtained using a transmission electron microscope (FEI Tecnai F20). The SEM images and EDS elemental mapping were made on a field emission scanning electron microscope (Zeiss Gemini500).

### MPA upconversion PL measurements

The fs laser system, including Ti:sapphire oscillator (Chameleon, Coherent; ~120 fs, 80 MHz, 800 nm), regenerative amplifier (Legend Elite, Coherent; 5 kHz, 800 nm), and optical parametric chirped-pulse amplifier (OPCPA, Topas-C, Coherent; 5 kHz, 400–2500 nm) was used as the excitation light. In all the experiments for the RPP bulks, the samples were rotated repeatedly to ensure the consistency between their *c*-axes and the polarization directions under all the excitation wavelengths, unless otherwise specified. The output laser of OPCPA was filtered by suitable long-pass filters to remove the light at undesired wavelengths. Two continuously variable neutral density filters were utilized to adjust the intensity of pump laser. The PL signals of RPPs were collected by an optical fiber and coupled to the spectrometer (QE Pro, Ocean Optics) in a reflection spectroscopy geometry. For the temperature-dependent PL measurements, the samples were put in a liquid nitrogen cryostat, and the temperature was set from 293 to 123 K with the cooling rate of 3.4 K/min.

### Time-dependent excitonic Bloch equations

The theoretical method to study the excitonic dipole behavior in the interaction of strong lasers with RPPs is based on solution of two-band semiconductor Bloch equations (SBEs) integrated with excitonic dipoles in two-band semiconductor. We consider a linearly polarized laser field propagating through crystal along the optical axis. In the independent-particle approximation, the SBEs with the velocity gauge read^75,76^

1 $$\hslash \frac{\partial }{\partial t}p\left(K,t\right)=-i\left[{{\rm{\varepsilon }}}_{c}^{K+A\left(t\right)}-{{\rm{\varepsilon }}}_{v}^{K+A\left(t\right)}-i\frac{\hslash }{{T}_{2}}\right]p\left(K,t\right)-{iE}(t)\cdot \left[{f}_{c}\left(K,t\right)-{f}_{v}\left(K,t\right)\right]{d}^{K+A(t)}$$

2 $$\hslash \frac{\partial }{\partial t}{f}_{c(v)}\left(K,t\right)=-2{\rm{Im}}\left[{d}^{K+A\left(t\right)}E\left(t\right){p}^{* }\left(K,t\right)\right]$$

Here, $p\left(K,t\right)$ is microscopic interband polarization between exciton corrected conduction and valence bands. *T*_2_ is the dephasing time. ${{\rm{\varepsilon }}}_{c}^{K+A\left(t\right)}$ and ${{\rm{\varepsilon }}}_{v}^{K+A\left(t\right)}$ are exciton corrected energy bands of the first conduction band and the highest valence band, respectively. ${d}^{K+A(t)}$ is the excitonic dipole matrix element. ${f}_{c}\left(K,t\right)$ and ${f}_{v}\left(K,t\right)$ are the populations of the first conduction and the upper valence bands, respectively. ${E\left(t\right)=E}_{0}f\left(t-{t}_{0}\right)\cos [\omega \left(t-{t}_{0}\right)+\varphi ]$ is the driving laser field. For $f\left(t-{t}_{0}\right)$, the Gaussian envelope is taken, and *t*_0_ = 288.75 fs. The full width at half maximum (FWHM) of both lasers is fixed at 120 fs, and *ω* is the frequency of the laser field. We keep the carrier-envelope phases (CEPs) *φ* = 0. *K* is the crystal momentum in a reciprocal reference frame moving with vector potential $A\left(t\right)=-{\int }_{0}^{t}E({t}^{{\prime} }){{dt}}^{{{\prime} }}$. We assume that electrons are initially filled in valence band. Then the macroscopic interband polarization *P*(*t*) of excitonic dipoles is given by

3 $$P\left(t\right)=\frac{d}{{dt}}{\int }_{{BZ}}\left[{d}^{K+A\left(t\right)}p\left(K,t\right)+c.c.\right]{dK}$$

Finally, the total intensity of MPA-induced PL spectra induced by BOED can be calculated by Fourier transforming the macroscopic interband polarization *P*(*t*) as

4 $$S\left(\omega \right)={\left|\frac{1}{\sqrt{2\pi }}{\int }_{0}^{T}P(t){e}^{-i\omega t}dt\right|}^{2}$$

In order to simplify the calculation, we consider only integration in each laser polarization direction.

### The anisotropy dispersion of energy bands and excitonic dipole matrix elements

The anisotropy dispersion of the lowest conduction band and the highest valence band for the three RPPs (Fig. S20) are calculated by using the Vienna Ab-initio Simulation Package (VASP) code^77–79^. Geometric optimizations of RPP crystals were done within generalized gradient approximation in the parametrization of Perdew-Burke-Ernzerhof (PBE)^78^. The energy cutoff was set to be 400 eV. Geometry structures are fully relaxed until the convergence criteria of energy and force are less than 10^–4^ eV and 0.03 eV Å^–1^, respectively. A Monkhorst-Pack mesh of 4×4×1 *k*-points is used in Brillouin zone for geometry optimizations and electronic structures are further calculated using the HSE06 hybrid function and considering spin-orbit interaction. The calculation results show that anisotropic dispersions of energy bands exhibit distinct four-fold symmetry (Fig. S20).

Moreover, we use the simplified model to generate the 2D energy bands. According to the ***k***·***p*** method^80^, the exciton corrected first conduction band (*ε*_c_) and the highest valence band (*ε*_*v*_) used in SBEs can be expressed as

5 $${\varepsilon }_{c}^{K+A(t)}={E}_{g}+\frac{{\hslash }^{2}{\left[K+A(t)\right]}^{2}}{2{m}_{0}}+\frac{{\hslash }^{2}{\left[K+A(t)\right]}^{2}{{|P|}}^{2}}{{{m}_{0}}^{2}{E}_{g}}$$

6 $${\varepsilon }_{v}^{K+A(t)}=\frac{{\hslash }^{2}{\left[K+A(t)\right]}^{2}}{2{m}_{0}}-\frac{{\hslash }^{2}{\left[K+A(t)\right]}^{2}{{|P|}}^{2}}{{{m}_{0}}^{2}{E}_{g}}$$

where *E*_g_ is the exciton corrected bandgap, *K* is the wave vector in reciprocal space, *A*(*t*) is the vector potential of the laser field, *P* is the momentum of electron, and *m*_0_ is the effective mass of electron. We define laser polarization direction along **Γ**–**X** direction as *θ* = 0. In order to get the energy bands of anisotropy distribution that matches the results from first-principles calculations, we introduce a distribution function to describe the anisotropy of momentum $P(\theta )={P}_{\theta =0}(1-\alpha |\sin \left(2\theta \right)|)$. Here, we set *α* = 0.7, and other parameters including lattice constant along laser polarization direction (*a*_*θ*=0_) used in simulation are shown in the following table.

**Table:** 

Parameters	CMPI	*i*-BMPB	F-BPB
*a*_*θ*=0_	8.7 Å	8.2 Å	8.3 Å
*E*_g_	2.02 eV	2.36 eV	2.82 eV
2 | *P*_*θ*=0_ | ^2^/*m*_0_	29 eV	33 eV	39 eV

Besides, the anisotropy ***k***-dependent excitonic dipole moments are obtained with first-order ***k***·**p** theory as

7 $${d}^{K+A(t)}=\frac{i{E}_{g}\,(1-\beta \,|\sin \left(2\theta \right)|)}{\left({\varepsilon }_{c}^{K+A\left(t\right)}\,-\,{\varepsilon }_{v}^{K+A(t)}\right)}$$

where *β* is set as 0.4, 0.4 and 0.45 for CMPI, *i*-BMPB and F-BPB, respectively.

## Supplementary information

supplementary material

## Data Availability

The data that support the findings of this study are available from the corresponding author upon reasonable request.
