# Supplementary material for: Giant anomalous exciton-multiphoton nonlinearities in layered hybrid perovskites
Source: Light Sci Appl. 2026 Aug 25;15:360. doi: 10.1038/s41377-026-02286-6 (PMC13503948; doi:10.1038/s41377-026-02286-6)
Supplement: Supplementary file 1 [file 41377_2026_2286_MOESM1_ESM.docx]

**Giant anomalous exciton-multiphoton nonlinearit****ies in layered hybrid perovskites**

Yanming Xu^1,2†^, Yi Liu^3†^, Chao Yu^4^^†^, Jinlong Xu^1,2,3^*, Wenqi Huang^1^, Weichu Mo^1^, Canhua Xu^1^, Zhihua Sun^3^*, Yantang Huang^1^, Xun Cao^2^*, Zhenda Xie^2^*, Ruifeng Lu^4^*

*^1^Fujian Key Laboratory of Quantum Information and Quantum Optics, College of Physics and Information Engineering, Fuzhou University, Fuzhou 350108, China*

*^2^National Laboratory of Solid State Microstructures, School of Electronic Science and Engineering, Nanjing University, Nanjing 210093, China*

*^3^**State Key Laboratory of Functional Crystals and Devices, Fujian Institute of Research on the Structure of Matter, Chinese Academy of Sciences, Fuzhou 350002, China*

*^4^**Institute of Ultrafast Optical Physics, Department of Applied Physics, Nanjing University of Science and Technology, Nanjing 210094, China*

^†^These authors contributed equally: Yanming Xu, Yi Liu, Chao Yu

*Correspondence: Jinlong Xu (longno.2@163.com); Zhihua Sun ([sunzhihua@fjirsm.ac.cn](mailto:sunzhihua@fjirsm.ac.cn)); Xun Cao ([caoxun@nju.edu.cn](mailto:caoxun@nju.edu.cn)); Zhenda Xie (xiezhenda@nju.edu.cn); Ruifeng Lu (rflu@njust.edu.cn)

**List of contents:**

**Supplementary Note 1:** Characterizations of the crystal structures.

**Supplementary Note 2:** Linear optical properties of the RPPs.

**Supplementary Note 3:** Exciton binding energies of the RPPs.

**Supplementary Note 4:** MPA upconversion PL spectra of the RPPs.

**Supplementary Note 5:** OA Z-scan measurements for the RPPs.

**Supplementary Note 6:** Polarized PL and absorbance measurements for the RPPs.

**Supplementary Note 7:** Characterizations of the RPP nanocomposites.

**Supplementary Note 8:** Comparison of the color gamut between the RPPs and sRGB.

**Supplementary Note 9:** Implementation methods of full-color MPA upconversion applications.

**Supplementary Note 1: Characterizations of the crystal structures**

Bruker D8 diffractometer with Mo *Kα* radiation (*λ* = 0.77 Å) was used to collect X-ray diffraction data. Crystal structures were solved by direct methods and confirmed by the full-matrix least-squares refinements on *F*^2^ using the *SHELXTL* software packing. All non-H atoms were refined anisotropically, and H atoms were generated by geometrical method and refined using the *Olex*2 software.

**Figure S1.** **Experimental and simulated powder X-ray diffraction (PXRD) patterns of the three RPPs.** (a) CMPI, (b) *i*-BMPB, and (c) F-BPB.

**Figure S2.** **Crystal structures, band structures, and QW structures of the three RPPs.** Left: crystal structure diagrams for top view in *bc* plane of (a) CMPI, (b) *i*-BMPB, and (c) F-BPB. Middle: Their DFT-calculated electronic band structures and the molecular local density of states. Right: Corresponding energy-level scheme evidencing type-I QW structures with high [potential](javascript:;) [barrier](javascript:;) heights.

**Figure S3.** **Comparison of the confinement potential for the three RPPs with other 2D perovskites.**

**Table S1.** Crystal data for the three RPPs.

|  | **CMPI** | ***i*-BMPB** | **F-BPB** |
| --- | --- | --- | --- |
| Empirical formula | C_15_H_38_I_7_N_3_Pb_2_ | C_10_H_36_Br_10_N_4_Pb_3_ | C_16_H_22_Br_4_F_2_N_2_Pb |
| Formula weight | 1563.16 | 1633.10 | 807.18 |
| Temperature (K) | 298 | 298 | 298 |
| Crystal system | Orthorhombic | Orthorhombic | Monoclinic |
| Space group | *Pna*2_1_ | *Ccce* | *C*2/*c* |
| *a* (Å) | 45.426(3) | 8.3668(6) | 33.478(7) |
| *b* (Å) | 8.8221(4) | 50.856(4) | 8.1466(16) |
| *c* (Å) | 8.8192(5) | 8.3721(7) | 8.4104(17) |
| *β* (º) | 90 | 90 | 96.137(2) |
| *V* (Å^3^) | 3534.3(3) | 3562.4(5) | 2280.7(8) |
| *ρ*_calca_ (Mg·m^-3^) | 2.938 | 3.045 | 2.351 |
| Z | 4 | 4 | 4 |
| *µ* (mm^-1^) | 15.637 | 25.367 | 14.424 |
| F(000) | 2736.0 | 2880.0 | 1488.0 |
| 2*θ* range (°) | 4.70 to 55.03 | 6.41 to 55.01 | 5.15 to 50 |
| Index ranges | -58≤h≤58, -11≤k≤ 10,  -11≤l≤11 | -10≤h≤10, -66≤k≤65,  -10≤l≤10 | -39≤h≤39, -9≤k≤9,  -10≤l≤ 10 |
| Reflections collected | 23962 | 12101 | 10071 |
| Independent reflections | 7325[*R*_int_=0.0359, *R*_sigma_=0.0391] | 2049[*R*_int_=0.0553, *R*_sigma_=0.0382] | 1968[*R*_int_=0.0492, *R*_sigma_=0.0354] |
| Data/restraints/parameters | 7325/119/250 | 2049/90/94 | 1968/0/116 |
| Goodness-of-fit on *F*^2^ | 1.029 | 1.096 | 1.076 |
| Final R indexes [*I*≥2*σ* (I)] | *R*_1_=0.0319, *wR*_2_=0.0713 | *R*_1_=0.0794, *wR*_2_=0.1838 | *R*_1_=0.0238, *wR*_2_=0.0516 |
| Final R indexes [all data] | *R*_1_=0.0473, *wR*_2_=0.0776 | *R*_1_=0.1207, *wR*_2_=0.2053 | *R*_1_=0.0298, *wR*_2_=0.0539 |
| *ρ*_calca_: cell desity; Z: molecule number in unit cell; *μ*: diffraction wavelength; F: electron number in unit cell; R: residual error. | | | |

**Table S2.** Confinement potential of the three RPPs compared with other reported RPPs.

| **Material** | ***E*_g_ (eV)** | ***V*_e_ (eV)** | **Ref.** |
| --- | --- | --- | --- |
| (BA)_2_SnI_4_ | 1.8 | 2 | 1 |
| (PMA)_2_PbI_4_ | 1.9 | 1.7 | 2 |
| (C_4_H_9_NH_3_)_2_PbI_4_ | 1.9 | 2.7 | 3 |
| (C_6_H_13_NH_3_)_2_PbI_4_ | 1.9 | 2.8 | 3 |
| (BA)_2_(MA)_2_Pb_3_I_10_ | 2.3 | 1.7 | 4 |
| (IA)_2_(MA)_2_Pb_3_Br_10_ | 2.3 | 2.5 | 5 |
| (PMA)_2_PbBr_4_ | 2.4 | 0.9 | 6 |
| (BA)_2_MAPb_2_I_7_ | 2.4 | 2.2 | 4 |
| (EA)_4_Pb_3_Br_10_ | 2.8 | 1.9 | 7 |
| (BA)_2_PbI_4_ | 2.8 | 1.8 | 1 |
| **(CA)_2_(MA)Pb_2_I_7_** | **2.0** | **5.6** | **This work** |
| **(*i*-BA)_2_(MA)_2_Pb_3_Br_10_** | **2.3** | **5.5** | **This work** |
| **(F-BEA)_2_PbBr_4_** | **2.8** | **4.5** | **This work** |
| *E*_g_: bandgap energy; *V*_e_: electron confinement potential. | | | |

**Supplementary Note 2: Linear optical properties of the RPPs**

**Figure S4.** **1PA-induced PL measurement for the three RPPs.** Left: 1PA PL spectra (*λ*_p_ = 405 nm) of (a) CMPI, (b) *i*-BMPB, and (c) F-BPB. Right: Logarithmic plot of the 1PA PL peak strength with respect to pump intensity of (a) CMPI, (b) *i*-BMPB, and (c) F-BPB. The 405-nm peak belongs to the excitation light.

**Supplementary Note 3: Exciton binding energies of the RPPs**

The exciton binding energies can be extracted from the temperature-dependent PL intensities (*λ*_p_ = 405 nm, CW laser). The PL intensities as a function of temperature are presented in Fig. S5, which can be fitted by the following equation^8^

$$\begin{aligned} I\left( T \right)=\frac{I_{0}}{1+Ae^{\left( -\frac{E_{b}}{k_{B}T} \right)}}\#\left( S1 \right) \end{aligned}$$

where *I*_0_ is the PL intensity at 0 K, *E*_b_ is the exciton binding energy, and *k*_B_ is the Boltzmann constant.

**Figure S5. Temperature-dependent PL intensities of CMPI, *i*-BMPB, and F-BPB.** The green dots are experimental data, and the solid red lines are the fitting of Eq. S1.

**Table S3.** Exciton binding energies of the three RPPs compared with other perovskites.

| **Material** | ***E*_g_ (eV)** | ***E*_b_ (meV)** | **Ref.** |
| --- | --- | --- | --- |
| MAPbI_3_ | 1.65 | 9±1 | 9 |
| MAPbI_3_ | 1.64 | 16±2 | 10 |
| MAPbI_3_ | 1.61 | 25±3 | 11 |
| MAPbI_3_ | 1.63 | 32±5 | 12 |
| MAPbBr_3_ | 2.36 | 65 | 13 |
| MAPbBr_3_ | 2.3 | 64 | 14 |
| MAPbBr_3_ | 2.4 | 80 | 15 |
| MAPbBr_3_ | 2.28±0.01 | 84±10 | 16 |
| MAPbBr_3_ | 2.31 | 37-60 | 17 |
| MAPbI_3-x_Cl_x_ | 1.65 | 55±20 | 18 |
| MAPbI_3-x_Cl_x_ | 1.61 | 62.3±8.9 | 19 |
| FAPbBr_3_ | 2.23 | 22 | 20 |
| FAPbBr_3_ | 2.27 | 22.1 | 21 |
| CsPbI_3_ | 1.72 | 15±1 | 22 |
| CsPbBr_3_ | 2.34 | 33±1 | 22 |
| CsPbBr3 | 2.35 | 25-50 | 23 |
| CsPbBr_3_ | 2.0 | 40 | 24 |
| CsPbI_2_Br | 1.91 | 22±3 | 22 |
| CsPbCl_3_ | 2.82 | 75 | 23 |
| Cs_0.05_(FA_0.83_MA_0.17_)_0.95_Pb(I_0.83_Br_0.17_)_3_ | 1.59-1.65 | 16-21 | 25 |
| Cs_0.1_(FA_0.675_MA_0.135_)_0.9_Pb(I_0.765_Br_0.235_)_3_ | 1.65-1.73 | 14-26 | 25 |
| (BA)_2_PbI_4_ | 2.79 | 249±5 | 26 |
| (BA)_2_PbI_4_ | 2.77 | 180 | 27 |
| (BA)_2_PbI_4_ | 2.8 | 260 | 28 |
| (BA)_2_(MA)_n-1_Pb_n_I_3n+1_ (n=1-5) | 1.85-2.42 | 380(n=1);270(n=2);220(n>2) | 29 |
| (BA)_2_(MA)_n-1_Pb_n_I_3n+1_ (n=1-5) | 1.85-2.54 | 470(n=1);250(n=2);175(n=3);150(n=4);125(n=5) | 30 |
| (PEA)_2_PbI_4_ | 2.61 | 220±6 | 26 |
| (PEA)_2_PbI_4_ | 2.36 | 190-250 | 22 |
| (PEA)_2_PbI_4_ | 2.57 | 160 | 27 |
| (PEA)_2_PbI_4_ | 2.34 | 250 | 31 |
| (PEA)_2_PbI_4_ | 2.56 | 200 | 28 |
| (BIA)_2_PbI_4_ | 2.4 | 150 | 32 |
| (BZA)_2_PbI_4_ | 2.2 | 244 | 32 |
| (R-MBA)_2_PbI_4_ | 2.8 | 260 | 33 |
| (BA_0.9_PEA_0.1_)_2_(MA)_n-1_Pb_n_I_3n+1_ | 1.66 | 256.5(n=2);243.5(n=3);61.7(n=∞) | 34 |
| (PEA)_2_PbBr_4_ | 2.85 | 347 | 35 |
| (PEA)_2_PbBr_4_ | 2.75 | 511 | 36 |
| (*p*-FPEA)_2_PbBr_4_ | 2.85 | 195 | 35 |
| (NEA)_2_PbBr_4_ | 2.5 | 249 | 36 |
| (BA)_2_SnI_4_ | 2.35 | 170±20 | 26 |
| (PEA)_2_SnI_4_ | 2.11 | 128±16 | 26 |
| (TEA)_2_SnI_4_ | 1.92(pristine)  1.96(with CA) | 263.3±30.1  467.6±43.3 | 37 |
| (R-MBA)_2_SnI_4_ | 2.6 | 250 | 33 |
| (R-MBA)_2_Pb_0.8_Sn_0.2_I_4_ | 2.4 | 230 | 33 |
| (R-MBA)_2_Pb_0.5_Sn_0.5_I_4_ | 2.4 | 230 | 33 |
| (R-MBA)_2_Pb_0.2_Sn_0.8_I_4_ | 2.5 | 240 | 33 |
| **(CA)_2_(MA)Pb_2_I_7_** | **2.0** | **309** | **This work** |
| **(*i*-BA)_2_(MA)_2_Pb_3_Br_10_** | **2.3** | **246** | **This work** |
| **(F-BEA)_2_PbBr_4_** | **2.8** | **332** | **This work** |
| *E*_g_: bandgap energy; *E*_b_: binding energy. | | | |

**Supplementary Note 4: MPA upconversion PL spectra of the RPPs**

**Figure S6. MPA upconversion PL spectra of the bulks at different NIR pump wavelengths for the three RPPs.** (a) CMPI, (b) *i*-BMPB, and (c) F-BPB.

**Figure S7.** **Photostability under intense fs-laser irradiation (*λ*_p_ = 1200 nm and *I*_p_ = 7.2 GW cm^-2^) for the three RPPs.** (a) CMPI, (b) *i*-BMPB, and (c) F-BPB.

**Figure S8.** **Long-term (after 15 months) stability measurement of the upconversion PL spectra (*λ*_p_ = 1200 nm and *I*_p_ = 7.2 GW cm^-2^) for the three RPPs.** (a) CMPI, (b) *i*-BMPB, and (c) F-BPB.

**Table S4.** Minimum pump intensity in 3PA-6PA processes of the three RPPs compared with other materials.

| **Material** | **MPA order** | ***I*_min_ (GW cm^-2^)** | **Ref.** |
| --- | --- | --- | --- |
| MAPbBr_3_ | 3 | ~23 | 38 |
| FAPbBr_3_ | 3 | ~8 | 39 |
| CsPbBr_3_ | 3 | ~12 | 38 |
| CsPbBr_3_ | 3 | 20 | 40 |
| MAPbBr_3_/(OA)_2_PbBr_4_ | 3 | ~13 | 38 |
| FAPbBr_3_/CsPbBr_3_ | 3 | ~8 | 39 |
| CsPbBr_3_@ZIF-8 | 3 | 7.8 | 40 |
| Zn_2_(SDC)_2_(An2Py) | 3 | ~11 | 41 |
| [Zn_2_(SDC)_2_(An2Py)]•perylene | 3 | ~8 | 41 |
| [Zn_2_(SDC)_2_(An2Py)]•anthracene | 3 | ~8 | 41 |
| SpL-3 | 3 | ~2 | 42 |
| Cs_3_Cu_2_I_5_ | 3 | ~48 | 43 |
| CsCu_2_I_3_ | 3 | ~66 | 43 |
| **(CA)_2_(MA)Pb_2_I_7_** | **3** | **1.1** | **This work** |
| **(*i*-BA)_2_(MA)_2_Pb_3_Br_10_** | **3** | **0.9** | **This work** |
| **(F-BEA)_2_PbBr_4_** | **3** | **1** | **This work** |
| MAPbBr_3_ | 4 | ~33 | 38 |
| FAPbBr_3_ | 4 | ~19 | 39 |
| CsPbBr_3_ | 4 | ~28 | 38 |
| CsPbBr_3_ | 4 | 18 | 40 |
| MAPbBr_3_/(OA)_2_PbBr_4_ | 4 | ~14 | 38 |
| FAPbBr_3_/CsPbBr_3_ | 4 | ~19 | 39 |
| ZJU-28⸧MAPbBr_3_ | 4 | 23.2 | 44 |
| CsPbBr_3_@ZIF-8 | 4 | 14 | 40 |
| Zn_2_(SDC)_2_(An2Py) | 4 | ~21 | 41 |
| [Zn_2_(SDC)_2_(An2Py)]•perylene | 4 | ~14 | 41 |
| [Zn_2_(SDC)_2_(An2Py)]•anthracene | 4 | ~10 | 41 |
| SpL-3 | 4 | ~8 | 42 |
| ZnO | 4 | 11.9 | 45 |
| **(CA)_2_(MA)Pb_2_I_7_** | **4** | **1.6** | **This work** |
| **(*i*-BA)_2_(MA)_2_Pb_3_Br_10_** | **4** | **1.1** | **This work** |
| **(F-BEA)_2_PbBr_4_** | **4** | **1.2** | **This work** |
| MAPbBr_3_ | 5 | ~85 | 38 |
| FAPbBr_3_ | 5 | ~71 | 39 |
| CsPbBr_3_ | 5 | ~85 | 38 |
| CsPbBr_3_ | 5 | 56 | 40 |
| MAPbBr_3_/(OA)_2_PbBr_4_ | 5 | ~67 | 38 |
| FAPbBr_3_/CsPbBr_3_ | 5 | ~71 | 39 |
| SpL-3 | 5 | ~12 | 42 |
| ZJU-28⸧MAPbBr_3_ | 5 | 36.3 | 44 |
| CsPbBr_3_@ZIF-8 | 5 | 39 | 40 |
| ZnO | 5 | 10.8 | 45 |
| **(*i*-BA)_2_(MA)_2_Pb_3_Br_10_** | **5** | **1.6** | **This work** |
| **(F-BEA)_2_PbBr_4_** | **5** | **1.3** | **This work** |
| MAPbCl_3_ | 6 | ~100 | 46 |
| CsPbCl_3_ | 6 | ~80 | 47 |
| SpL-3 | 6 | ~22 | 42 |
| **(F-BEA)_2_PbBr_4_** | **6** | **1.8** | **This work** |
| *I*_min_: the minimum pump intensity. | | | |

**Supplementary Note 5: OA Z-scan measurements for the RPPs**

As a nonlinear optical effect, the MPA processes can be described by the following expression^48^

$$\begin{aligned} \frac{dI\left( z \right)}{d\left( z \right)}=-\alpha I\left( z \right)-\beta I^{2}\left( z \right)-\gamma I^{3}\left( z \right)-\delta I^{4}\left( z \right)-\varphi I^{5}\left( z \right)-\eta I^{6}\left( z \right)-\cdots\#\left( S2 \right) \end{aligned}$$

where *I*(*z*) is the local intensity of incident laser beam propagating along the *z*-axis, and *z* is the propagation distance of light in the medium. The parameters *α*, *β*, *γ*, *δ*, *φ*, and *η* are one-, two-, three-, four-, five-, and six-photon nonlinear absorption coefficients, respectively. Here, 6PA nonlinear optical process is given as an example. Suppose at a certain photon frequency *ν*, only the 6PA process satisfying Eq. S2 is available, then the following relation can be obtained

$$\begin{aligned} \frac{dI\left( z \right)}{dz}=-\eta I^{6}\left( z \right)\#\left( S3 \right) \end{aligned}$$

and its solution is

$$\begin{aligned} I\left( z \right)=\frac{I_{0}}{\left( 1+5\eta zI_{0}^{5} \right)^{\frac{1}{5}}}\#\left( S4 \right) \end{aligned}$$

when *z* = *L*_0_, the nonlinear transmittance *T* of a six-photon absorbing medium can be expressed as

$$\begin{aligned} T=\frac{I\left( L_{0} \right)}{I_{0}}=\frac{1}{\left( 1+5\eta zI_{0}^{5} \right)^{\frac{1}{5}}}\#\left( S5 \right) \end{aligned}$$

where *I*_0_ is the intensity of incident laser and *L*_0_ is the optical path length.

Thus, the *η* value of a given medium can be determined experimentally by measuring the transmittance *T* at a given incident intensity *I*_0_. Note that, the value of *I*_0_ varies with the position *z* along the *z*-axis direction.

Further, for the crystal flakes, the 6PA cross-section *σ*_6_ can be calculated according to the following equation

$$\begin{aligned} \sigma_{6}=\frac{\left( h\nu\right)^{5}\eta}{N_{\mathrm{crystal}}}\#\left( S6 \right) \end{aligned}$$

where *hν* is the photon energy of incident light, and *N*_crystal_ is the molecular density of hybrid perovskites (in units of cm^-3^).

While for the DCM solution of NCs, the 6PA cross-sections can be calculated as follows

$$\begin{aligned} \sigma_{6}=\frac{\left( h\nu\right)^{5}\eta}{N_{A}d_{0}\times{10}^{-3}}\#\left( S7 \right) \end{aligned}$$

here, *N*_A_ is Avogadro’s number, and *d*_0_ is the molar concentration of the NCs (in units of mol L^-1^).

Similarly, the absorption coefficients and cross-sections for 2PA-5PA, can be determined experimentally and calculated based on this theory.

The schematic diagram of the setup for OA Z-scan measurements is in Fig. S9. The incident laser beam was divided into two parts by a beam splitter. One part was directed into a power detector (*D*_R_) as the reference, while the other part was focused onto the crystal flakes (the thickness ~0.2 mm) or the NC solution (in a 1-mm-thick quartz cuvette) by a circular lens with the focal length of 200 mm, and the beam transmitted through the samples was detected by another power detector (*D*_S_) as the signal. The incident beam was propagating along the crystallographic *a*-axis and the samples moved along the propagating direction of the beam, i.e., *z*-axis. The transmittance was recorded as a function of the sample position (*z*). As the incident power can be regarded as a constant, the sample will be subjected to various peak intensity *I*(*z*) at different position (*z*) and subsequently the change of transmittance. The diameters of the laser beam at different position (*z*) were measured through knife-edge scans along the *x*-axis direction of the cross-sectional plane.

**Figure S9. Schematic diagram of the OA Z-scan setup.** *D*_S_ and *D*_R_ denote the signal power detector and reference power detector, respectively.

**Figure S10.** **Normalized transmittance as a function of pump intensity at different NIR pump wavelengths for the bulks.** (a) CMPI (2PA-4PA), (b) *i*-BMPB (3PA-5PA), and (c) F-BPB (4PA-6PA). The green hollow dots are experimental data, and the red lines are the fitting to the data.

To rule out the contribution from the solvent to the measured nonlinear absorption, an OA Z-scan measurement was performed on the pure DCM solvent under identical conditions. No nonlinear absorption signals were detected from the solvent within the intensity range applied in this work (Fig. S11), confirming that the observed MPA responses originate exclusively from the RPP NCs.

**Figure S11.** **Z-scan curves measured at different NIR pump wavelengths for the NCs (dispersed in DCM) and the pure DCM.** (a) CMPI (2PA-4PA), (b) *i*-BMPB (3PA-5PA), and (c) F-BPB (4PA-6PA).

**Figure S12.** **Normalized transmittance as a function of pump intensity at different NIR pump wavelengths for the NCs.** (a) CMPI (2PA-4PA), (b) *i*-BMPB (3PA-5PA), and (c) F-BPB (4PA-6PA). The green hollow dots are experimental data, and the red lines are the fitting to the data.

**Figure S13. Comparison of the measured 5PA and 6PA absorption cross-sections of the two RPP NCs with reported representative results.** References: MAPbBr_3_^38^, CsPbBr_3_^38^, CsPbBr_2.7_I_0.3_^51^, Cs_2_TeCl_6_^53^, MAPbBr_3_/(OA)_2_PbBr_4_^38^, FAPbBr_3_/CsPbBr_3_^39^, ZJU-28⊃MAPbBr_3_^44^, CsPbBr_3_@ZIF-8^40^, IPPS^54^, SpL-3^42^, and ZnO^45^.

**Table S5.** Multiphoton absorption coefficients and cross-sections of the three RPPs compared with other materials.

| **Material** | ***E*_g_ (eV)** | ***λ*_p_ (nm)** | **Absorption coefficient** | **Absorption cross-section** | **Ref.** |
| --- | --- | --- | --- | --- | --- |
| **3PA process** | | | | | |
| MAPbI_3_ | 1.49 | 1600/2100 | *γ* =3.3×10^-4^/4.8×10^-4^ cm^3^GW^-2^ | / | 49 |
| MAPbBr_3_ | 2.36 | 1050-1500 | / | *σ*_3_=(0.39-3.2)×10^-78^ cm^6^s^2^photon^-2^ | 38 |
| FAPbBr_3_ | 2.18 | 1300 | / | *σ*_3_=7.7×10^-78^ cm^6^s^2^photon^-2^ | 39 |
| CsPbBr_3_ | 2.39 | 1050-1500 | / | *σ*_3_=(0.27-2.4)×10^-73^ cm^6^s^2^photon^-2^ | 38 |
| CsPbBr_3_ | 2.2 | 1300 | / | *σ*_3_=4.1×10^-76^ cm^6^s^2^photon^-2^ | 40 |
| Cs_4_PbBr_6_ | 4 | 900-1200 | *γ*=7.2-8.4 cm^3^GW^-2^ | *σ*_3_=(0.96-1.5)×10^-72^ cm^6^s^2^photon^-2^ | 50 |
| Cs_4_PbBr_6_ | 2.46 | 900-1200 | *γ*=0.7-0.8 cm^3^GW^-2^ | *σ*_3_=(0.77-1.1)×10^-72^ cm^6^s^2^photon^-2^ | 50 |
| CsPbBr_2.7_I_0.3_ | 2.6/2.4 | 800 | / | *σ*_3_=2.0×10^-75^/2.3×10^-74^ cm^6^s^2^photon^-2^ | 51 |
| (Cs_0.06_FA_0.79_MA_0.15_)Pb(I_0.85_Br_0.15_)_3_ | 1.58 | 790/1200 | *γ*=691-900/2.8-26.5 cm^3^GW^-2^ | / | 52 |
| CsPbCl_3_ | 2.91 | 1200 | *γ*=8.9×10^-2^ cm^3^GW^-2^ | / | 47 |
| Cs_2_TeCl_6_ | 2.69 | 800 | / | *σ*_3_=1.4×10^-78^ cm^6^s^2^photon^-2^ | 53 |
| MAPbBr_3_/(OA)_2_PbBr_4_ | / | 1050-1500 | / | *σ*_3_=(0.069-1.4)×10^-73^ cm^6^s^2^photon^-2^ | 38 |
| FAPbBr_3_/CsPbBr_3_ | / | 1300 | / | *σ*_3_=5.9×10^-77^ cm^6^s^2^photon^-2^ | 39 |
| ZJU-28⊃MAPbBr_3_ | 2.38 | 1440 | / | *σ*_3_=2.7×10^-74^ cm^6^s^2^photon^-2^ | 44 |
| CsPbBr_3_@ZIF-8 | 2.3 | 1300 | / | *σ*_3_=1.1×10^-73^ cm^6^s^2^photon^-2^ | 40 |
| Zn_2_(SDC)_2_(An2Py) | 2.7 | 950-1400 | / | *σ*_3_=(0.02-4.6)×10^-79^ cm^6^s^2^photon^-2^ | 41 |
| [Zn_2_(SDC)_2_(An2Py)]•perylene | 2.6 | 950-1400 | / | *σ*_3_=(0.08-4.3)×10^-79^ cm^6^s^2^photon^-2^ | 41 |
| [Zn_2_(SDC)_2_(An2Py)]•anthracene | 2.6 | 950-1400 | / | *σ*_3_=(0.1-5.0)×10^-79^ cm^6^s^2^photon^-2^ | 41 |
| IPPS | 2.5 | 1197 | *γ*=(1.2±0.18)×10^-4^ cm^3^GW^2^ | *σ*_3_=3.7×10^-80^ cm^6^s^2^photon^-2^ | 54 |
| SpL-3 | 2.6 | 940 | / | *σ*_3_=2.4×10^-74^ cm^6^s^2^photon^-2^ | 42 |
| Cs_3_Cu_2_I_5_ | 2.8 | 800 | *γ*=4.7×10^-5^ cm^3^GW^-2^ | / | 43 |
| CsCu_2_I_3_ | 2.1 | 800 | *γ*=2.7×10^-4^ cm^3^GW^-2^ | / | 43 |
| ZnO | 2.2 | 800-1100 | / | *σ*_3_=(0.2-7.0)×10^-79^ cm^6^s^2^photon^-2^ | 45 |
| CdS | 2.8 | 900-1000 | / | *σ*_3_=10^-79^ cm^6^s^2^photon^-2^ | 55 |
| ZnS | 2.8 | 780 | *γ*=2.4×10^-2^ cm^3^GW^-2^ | *σ*_3_=2.7×10^-78^ cm^6^s^2^photon^-2^ | 56 |
| GaS | 2.3 | 1500 | *γ*=4.9 cm^3^GW^-2^ | *σ*_3_=8.2×10^-80^ cm^6^s^2^photon^-2^ | 57 |
| GaN | 3.4 | 760-1000 | *γ*=(0.65-3.5)×10^-3^ cm^3^GW^-2^ | / | 58 |
| MoS_2_ | 2.7 | 1300 | *γ*=2.2 cm^3^GW^-2^ | / | 59 |
| **(CA)_2_(MA)Pb_2_I_7_**  **Bulk crystals**  **Nano crystals** | **2.0** | **1550** | ***γ*=13.2 cm^3^GW^-2^**  ***γ*=0.3 cm^3^GW^-2^** | ***σ*_3_=2.0×10^-76^ cm^6^s^2^photon^-2^**  ***σ*_3_=1.7×10^-72^ cm^6^s^2^photon^-2^** | **This work** |
| **(*i*-BA)_2_(MA)_2_Pb_3_Br_10_**  **Bulk crystals**  **Nano crystals** | **2.3** | **1200** | ***γ*=214.8 cm^3^GW^-2^**  ***γ*=0.60 cm^3^GW^-2^** | ***σ*_3_=5.1×10^-75^ cm^6^s^2^photon^-2^**  ***σ*_3_=6.3×10^-72^ cm^6^s^2^photon^-2^** | **This work** |
| **(F-BEA)_2_PbBr_4_**  **Bulk crystals**  **Nano crystals** | **2.8** | **1200** | ***γ*=15.5 cm^3^GW^-2^**  ***γ*=0.98 cm^3^GW^-2^** | ***σ*_3_=2.6×10^-76^ cm^6^s^2^photon^-2^**  ***σ*_3_=5.3×10^-72^ cm^6^s^2^photon^-2^** | **This work** |
| **4PA process** | | | | | |
| MAPbBr_3_ | 2.36 | 1550-2000 | / | *σ*_4_=(0.043-3.6)×10^-104^ cm^8^s^3^photon^-3^ | 38 |
| FAPbBr_3_ | 2.18 | 2000 | / | *σ*_4_=1.6×10^-107^ cm^8^s^3^photon^-3^ | 39 |
| CsPbBr_3_ | 2.39 | 1550-2000 | / | *σ*_4_=(0.013-1.3)×10^-103^ cm^8^s^3^photon^-3^ | 38 |
| CsPbBr_3_ | 2.2 | 1800 | / | *σ*_4_=1.5×10^-106^ cm^8^s^3^photon^-3^ | 40 |
| CsPbCl_3_ | 2.91 | 1600 | *δ*=1.1×10^-4^ cm^5^GW^-3^ | / | 47 |
| CsPbBr_2.7_I_0.3_ | 2.6/2.4 | 1600 | / | *σ*_4_=0.13×10^-104^/2.06×10^-104^ cm^8^s^3^photon^-3^ | 51 |
| Cs_4_PbBr_6_ | 4 | 1300-1500 | *δ*=15.5-33.3 cm^5^GW^-3^ | *σ*_4_=(1.5-4.0)×10^-100^ cm^8^s^3^photon^-3^ | 50 |
| Cs_4_PbBr_6_ | 2.46 | 1300-1500 | *δ*=13.2-26 cm^5^GW^-3^ | *σ*_4_=(1.2-2.5)×10^-99^ cm^8^s^3^photon^-3^ | 50 |
| Cs_2_TeCl_6_ | 2.69 | 1560 | / | *σ*_4_=6.5×10^-110^ cm^8^s^3^photon^-3^ | 53 |
| MAPbBr_3_/(OA)_2_PbBr_4_ | / | 1550-2000 | / | *σ*_4_=(0.023-2.6)×10^-103^ cm^8^s^3^photon^-3^ | 38 |
| FAPbBr_3_/CsPbBr_3_ | / | 2000 | / | *σ*_4_=1.2×10^-106^ cm^8^s^3^photon^-3^ | 39 |
| ZJU-28⊃MAPbBr_3_ | 2.38 | 1880 | / | *σ*_4_=5.3×10^-105^ cm^8^s^3^photon^-3^ | 44 |
| CsPbBr_3_@ZIF-8 | 2.3 | 1800 | / | *σ*_4_=7.5×10^-104^ cm^8^s^3^photon^-3^ | 40 |
| Zn_2_(SDC)_2_(An2Py) | 2.7 | 1450-1500 | / | *σ*_4_=(1.7-3.2)×10^-110^ cm^8^s^3^photon^-3^ | 41 |
| [Zn_2_(SDC)_2_(An2Py)]•perylene | 2.6 | 1450-1500 | / | *σ*_4_=(0.7-1.1)×10^-109^ cm^8^s^3^photon^-3^ | 41 |
| [Zn_2_(SDC)_2_(An2Py)]•anthracene | 2.6 | 1450-1500 | / | *σ*_4_=(1.3-1.8)×10^-109^ cm^8^s^3^photon^-3^ | 41 |
| SpL-3 | 2.6 | 1440 | / | *σ*_4_=5.1×10^-107^ cm^8^s^3^photon^-3^ | 42 |
| ZnO | 2.2 | 1150-1450 | / | *σ*_4_=(0.4-4.8)×10^-107^ cm^8^s^3^photon^-3^ | 45 |
| **(CA)_2_(MA)Pb_2_I_7_**  **Bulk crystals**  **Nano crystals** | **2.0** | **2300** | ***δ*=2.3 cm^5^GW^-3^**  ***δ*=3.9×10^-2^ cm^5^GW^-3^** | ***σ*_4_=1.3×10^-105^ cm^8^s^3^photon^-3^**  ***σ*_4_=8.5×10^-102^ cm^8^s^3^photon^-3^** | **This work** |
| **(*i*-BA)_2_(MA)_2_Pb_3_Br_10_**  **Bulk crystals**  **Nano crystals** | **2.3** | **1750** | ***δ*=14.7 cm^5^GW^-3^**  ***δ*=1.1×10^-2^ cm^5^GW^-3^** | ***σ*_4_=1.8×10^-104^ cm^8^s^3^photon^-3^**  ***σ*_4_=6.2×10^-102^ cm^8^s^3^photon^-3^** | **This work** |
| **(F-BEA)_2_PbBr_4_**  **Bulk crystals**  **Nano crystals** | **2.8** | **1550** | ***δ*=6.0 cm^5^GW^-3^**  ***δ*=0.14 cm^5^GW^-3^** | ***σ*_4_=7.8×10^-105^ cm^8^s^3^photon^-3^**  ***σ*_4_=5.8×10^-102^ cm^8^s^3^photon^-3^** | **This work** |
| **5PA process** | | | | | |
| MAPbBr_3_ | 2.36 | 2050-2300 | / | *σ*_5_=(0.05-2.9)×10^-136^ cm^10^s^4^photon^-4^ | 38 |
| FAPbBr_3_ | 2.18 | 2300 | / | *σ*_5_=1.2×10^-139^ cm^10^s^4^photon^-4^ | 39 |
| MAPbCl_3_ | 2.9 | 1800 | *φ*=3.0×10^-8^ cm^7^GW^-4^ | / | 46 |
| CsPbBr_3_ | 2.39 | 2050-2300 | / | *σ*_5_=(0.02-1.2)×10^-135^ cm^10^s^4^photon^-4^ | 38 |
| CsPbBr_3_ | 2.2 | 2200 | / | *σ*_5_=8.0×10^-139^ cm^10^s^4^photon^-4^ | 40 |
| CsPbCl_3_ | 2.91 | 1800 | *φ*=2.3×10^-7^ cm^7^GW^-4^ | / | 47 |
| CsPbBr_2.7_I_0.3_ | 2.6/2.4 | 2200 | / | *σ*_5_=0.12×10^-136^/1.5×10^-136^ cm^10^s^4^photon^-4^ | 51 |
| Cs_2_TeCl_6_ | 2.69 | 1640 | / | *σ*_5_=2.4×10^-142^ cm^10^s^4^photon^-4^ | 53 |
| MAPbBr_3_/(OA)_2_PbBr_4_ | / | 2050-2300 | / | *σ*_5_=(0.032-2.2)×10^-135^ cm^10^s^4^photon^-4^ | 38 |
| FAPbBr_3_/CsPbBr_3_ | / | 2300 | / | *σ*_5_=9.3×10^-139^ cm^10^s^4^photon^-4^ | 39 |
| ZJU-28⊃MAPbBr_3_ | 2.38 | 2100 | / | *σ*_5_=1.0×10^-135^ cm^10^ s^4^ photon^-4^ | 44 |
| CsPbBr_3_@ZIF-8 | 2.3 | 2200 | / | *σ*_5_=6.2×10^-136^ cm^10^ s^4^ photon^-4^ | 40 |
| IPPS | 2.5 | 2100 | *φ*=(2.2±0.32)×10^-11^ cm^7^GW^-4^ | *σ*_5_=1.9×10^-143^ cm^10^ s^4^ photon^-4^ | 54 |
| SpL-3 | 2.6 | 1540 | / | *σ*_5_=9.3×10^-137^ cm^10^ s^4^ photon^-4^ | 42 |
| ZnO | 2.2 | 1500-1600 | / | *σ*_5_=(0.3-6.5)×10^-136^ cm^10^ s^4^ photon^-4^ | 45 |
| **(*i*-BA)_2_(MA)_2_Pb_3_Br_10_**  **Bulk crystals**  **Nano crystals** | **2.3** | **2300** | ***φ*=9.4cm^7^ GW^-4^**  ***φ*=1.8×10^-3^ cm^7^GW^-4^** | ***σ*_5_=4.5×10^-133^ cm^10^s^4^photon^-4^**  ***σ*_5_=3.9×10^-131^ cm^10^s^4^photon^-4^** | **This work** |
| **(F-BEA)_2_PbBr_4_**  **Bulk crystals**  **Nano crystals** | **2.8** | **1850** | ***φ*=1.1cm^7^ GW^-4^**  ***φ*=7.5×10^-3^ cm^7^GW^-4^** | ***σ*_5_=9.1×10^-134^ cm^10^s^4^photon^-4^**  ***σ*_5_=2.0×10^-131^ cm^10^s^4^photon^-4^** | **This work** |
| **6PA process** | | | | | |
| MAPbCl_3_ | 2.9 | 2400 | *η*=9×10^-10^ cm^9^GW^-5^ | / | 46 |
| CsPbCl_3_ | 2.91 | 2200 | *η*=1.1×10^-10^ cm^9^GW^-5^ | / | 47 |
| Cs_2_TeCl_6_ | 2.69 | 1980 | / | *σ*_6_=1.9×10^-174^ cm^12^s^5^photon^-5^ | 53 |
| SpL-3 | 2.6 | 1820 | / | *σ*_6_=8.7×10^-169^ cm^12^s^5^photon^-5^ | 42 |
| **(F-BEA)_2_PbBr_4_**  **Bulk crystals**  **Nano crystals** | **2.8** | **2300** | ***η*=0.14 cm^9^GW^-5^**  ***η*=1.0×10^-4^ cm^9^GW^-5^** | ***σ*_6_=4.2×10^-163^ cm^12^s^5^photon^-5^**  ***σ*_6_=9.5×10^-161^ cm^12^s^5^photon^-5^** | **This work** |
| *E*_g_: bandgap energy; *λ*_p_: pump wavelength; *β*, *γ*, *δ*, *φ*, and *η*: 2PA-6PA absorption coefficient, respectively; σ_2_-σ_6_: 2PA-6PA absorption cross-section, respectively. | | | | | |

**Supplementary Note 6: Polarized PL and absorbance measurements for the RPPs**

The schematic diagram of the setup for polarized MPA-induced PL measurements is plotted in Fig. 2a in the main text. The polarization of the incident electric field was altered through rotating a half-wave plate, and the electric field polarization was set parallel to the *c*-axis of the crystal flakes initially. Note that, the relative angle (*θ*) between the electric field polarization and the crystallographic *c*-axis is twice the rotated angle of the half-wave plate. For the polarized absorbance measurements, a power detector placed after the crystal flakes was utilized to detect the transmitted power *P*(*θ*) once the electric field polarization was altered. Since the incident power detected is *P*_0_, the absorbed power at different relative angle (*θ*) could be calculated as *P*_0_ *-* *P*(*θ*). To avoid the scattering from crystal flakes’ surface, the samples were placed as close as to the power detector.

**Figure S14. Representative polar plots demonstrate the orientation evolutions of 1PA PL (*λ*_p_ = 400 nm) in the three RPPs.** (a) CMPI, (b) *i*-BMPB, and (c) F-BPB.

**Figure S15. False-color mapping of crystallographic orientation-dependent MPA PL spectra.** (a) Upper: 3PA of CMPI (*λ*_p_ = 1550 nm); lower: 4PA of CMPI (*λ*_p_ = 2300 nm). (b) Upper: 4PA of *i*-BMPB (*λ*_p_ = 1800 nm); lower: 5PA of *i*-BMPB (*λ*_p_ = 2300 nm). (c) Upper: 5PA of F-BPB (*λ*_p_ = 1800 nm); lower: 6PA of F-BPB (*λ*_p_ = 2300 nm).

**Figure S16. Representative polar plots demonstrate the orientation evolutions in *i*-BMPB.** (a) Orientation evolutions of 2PA absorbance (*λ*_p_ = 800 nm). (b) Orientation evolutions of 5PA absorbance (*λ*_p_ = 2300 nm). (c) Left: orientation evolutions of 4PA PL (*λ*_p_ = 1800 nm); right: orientation evolutions of the corresponding absorbance. The green hollow dots are experimental data, and the red lines are the simulation results.

**Figure S17. Representative polar plots demonstrate the orientation evolutions in CMPI.** (a) Left: orientation evolutions of 2PA PL (*λ*_p_ = 800 nm); right: orientation evolutions of the corresponding absorbance. (b) Left: orientation evolutions of 3PA PL (*λ*_p_ = 1550 nm); right: orientation evolutions of the corresponding absorbance. (c) Left: orientation evolutions of 4PA PL (*λ*_p_ = 2300 nm); right: orientation evolutions of the corresponding absorbance. (d) Orientation evolutions under different pump wavelengths at a constant pump intensity of 5.1 GW cm^-2^. The red hollow dots are experimental data, and the red lines are the simulation results.

**Figure S18. Representative polar plots demonstrate the orientation evolutions in F-BPB.** (a) Left: orientation evolutions of 2PA PL (*λ*_p_ = 800 nm); right: orientation evolutions of the corresponding absorbance. (b) Left: orientation evolutions of 5PA PL (*λ*_p_ = 1800 nm); right: orientation evolutions of the corresponding absorbance. (c) Left: orientation evolutions of 6PA PL (*λ*_p_ = 2300 nm); right: orientation evolutions of the corresponding absorbance. (d) Orientation evolutions under different pump wavelengths at a constant pump intensity of 5.1 GW cm^-2^. The blue hollow dots are experimental data, and the red lines are the simulation results.

**Figure S19.** **Extinction ratios (*ε*) as a function of the pump intensity in different MPA processes for the two RPPs.** (a) CMPI and (b) F-BPB.

**Figure S20. Calculated 3D energy band structure (upper) and the extracted energy difference between the CBM and VBM (lower) of the three RPPs.** (a) CMPI, (b) *i*-BMPB, and (c) F-BPB.

**Supplementary Note 7: Characterizations of the RPP nanocomposites**

**Figure S21. SEM image of the composite and the corresponding EDS elemental mapping for the three RPPs.** (a) CMPI/F-BPB (C/F) and (b) *i*-BMPB/F-BPB (B/F).

**Figure S22. Time-resolved PL curves for** **the three NCs and their nanocomposites.** (a) CMPI, (b) *i*-BMPB, (c) F-BPB, (d) CMPI of C/B, (e) *i*-BMPB of C/B, (f) CMPI of C/F, (g) F-BPB of C/F, (h) *i*-BMPB of B/F, and (i) F-BPB of B/F. The green hollow dots are experimental data, and the red lines are the exponential fitting to the data.

**Figure S23. Comparison of PL lifetimes extracted from TRPL measurements for the three NCs and their nanocomposites.**

**Figure S24. Stability measurement for the C/F composites (*λ*_p_ = 2300 nm and *I*_p_ = 9.2 GW cm^-2^).** (a) PL stability of C/F for magenta emission under fs laser pumping for 90 min. (b) Time dependent PL emission spectra for C/F, where the PL peaks located at ~430 and 618 nm correspond to F-BPB and CMPI, respectively.

**Supplementary Note 8: Comparison of the color gamut between the RPPs and sRGB**

The CIE1931 chromaticity color diagram of the RPP NCs and the standard RGB (sRGB)^60^ is shown in Fig. 4f in the main text, and the corresponding CIE1931 chromaticity coordinates (*x*, *y*) are listed in Table S6. To compare the color gamut of the RPP NCs with the sRGB, the CIE1976 chromaticity coordinates (*u*′, *v*′) listed in Table S7 can be acquired from the following equation^61^

$$\begin{aligned} u^{'}=\frac{4x}{-2x+12y+3},v^{'}=\frac{9y}{-2x+12y+3}\#\left( S8 \right) \end{aligned}$$

Since three coordinates constitute a triangle in the CIE color diagram, all the colors inside the triangle can be obtained by mixing two or three of the RGB colors with proper ratios. The area of the RGB triangle (*A*) can be calculated according to the following equation^62^

$$\begin{aligned} A=\frac{\left( u_{r}^{'}-u_{b}^{'} \right)\left( v_{g}^{'}-v_{b}^{'} \right)-\left( u_{g}^{'}-u_{b}^{'} \right)\left( v_{r}^{'}-v_{b}^{'} \right)}{2}\#\left( S9 \right) \end{aligned}$$

The areas of the RPP NCs and the sRGB are calculated to be 0.0699 and 0.0649, respectively, hence the RPP NCs can achieve a color gamut 7.7% larger than the sRGB.

**Table S6.** CIE1931 chromaticity coordinates (*x*, *y*) of the RPP NCs and sRGB.

| **Sample** | | ***x*** | ***y*** |
| --- | --- | --- | --- |
| RPP NCs | R | 0.696 | 0.304 |
|  | G | 0.236 | 0.689 |
|  | B | 0.179 | 0.108 |
| sRGB | R | 0.640 | 0.330 |
|  | G | 0.300 | 0.600 |
|  | B | 0.150 | 0.060 |

**Table S7.** CIE1976 chromaticity coordinates (*u*′, *v*′) of the RPP NCs and sRGB.

| **Sample** | | ***u′*** | ***v′*** |
| --- | --- | --- | --- |
| RPP NCs | R | 0.529 | 0.520 |
|  | G | 0.087 | 0.574 |
|  | B | 0.182 | 0.246 |
| sRGB | R | 0.451 | 0.523 |
|  | G | 0.125 | 0.563 |
|  | B | 0.175 | 0.158 |

**Supplementary Note 9: Implementation methods of full-color MPA upconversion applications**

For the full-color upconversion displays, the patterns were drawn in advance on the aluminum plate’s surface with the identical NC solution as previously used in the full-color fluorescence emission experiments. After evaporation of the DCM solvent, the NIR-III/IV fs laser beam was focused by a circular lens with the focal length of 200 mm onto the aluminum plate’s surface. The aluminum plate was driven directionally to enable the focused laser beam to scan the aluminum plate’s surface point by point and line by line. The built-in camera in a cell phone fixed on top of the aluminum plate and working in the delay shooting mode (delay time is 32 s) was utilized to take photographs.

For the demonstration of decoding information, a quick-response (QR) code was drawn in advance on the aluminum plate’s surface with the NC solution of *i*-BMPB. The way of taking photographs was identical to that in the full-color upconversion displays. As a reference, the photographs were taken under the irradiation of continuous-wave (CW) laser (*λ*_p_ = 650, 532, and 444 nm) and the nanosecond (ns) laser (*λ*_p_ = 2000 nm) as well. In the case of CW laser irradiation, the laser beam was expanded by a circular lens with the focal length of 30 mm to ensure the laser spot could cover the whole pattern drawn beforehand, and the camera was working in the normal shooting mode. While in the case of ns-laser irradiation, the methods of focusing the laser beam onto the aluminum plate’s surface and taking photographs were as same as the fs-laser irradiation condition. The incident mean power was set approximately equal (~60 mW) when different lasers were employed as the irradiation sources.

For the demonstration of higher-confidentiality dual decryption, the images of three figures were drawn in advance on the aluminum plate’s surface with the NC solution of CMPI, *i*-BMPB, and F-BPB, respectively. To obtain the individual figures in red, green, and blue, respectively, the filters centered at 632, 532, and 442 nm were put in front of the camera, respectively.

**References**

1. Mitzi, D. B. Synthesis, crystal structure, and optical and thermal properties of (C_4_H_9_NH_3_)_2_MI_4_ (M = Ge, Sn, Pb). *Chem. Mater.* **8**, 791-800 (1996).
2. Papavassiliou, G. C. et al. A. Preparation and characterization of [C_6_H_5_CH_2_NH_3_]_2_PbI_4_, [C_6_H_5_CH_2_CH_2_SC(NH_2_)_2_]_3_PbI_5_ and [C_10_H_7_CH_2_NH_3_]PbI_3_ organic-inorganic hybrid compounds. *Z. Naturforsch.* **54b**, 1405-1409 (1999).
3. Traore, B. et al. Composite nature of layered hybrid: assessment on quantum and dielectric confinements and band alignment. *ACS Nano* **12**, 3321-3332 (2018).
4. Stoumpos, C. C. et al. Ruddlesden-Popper hybrid lead iodide perovskite 2D homologous semiconductors. *Chem. Mater.* **28**, 2852-2867 (2016).
5. Li, M. et al. Giant and broadband multiphoton absorption nonlinearities of a 2D organometallic perovskite ferroelectric. *Adv. Mater.* **32**, 2002972 (2020).
6. Kawano, N. et al. Effects of organic moieties on luminescence properties of organic-inorganic layered perovskite-type compounds. *J. Phys. Chem. C* **118**, 9101-9106 (2014).
7. Mao, L. et al. Tunable white-light emission in single-cation-templated threelayered 2D perovskites (CH_3_CH_2_NH_3_)_4_Pb_3_Br_10-x_Cl_x_. *J. Am. Chem. Soc.* **139**, 11956-11963 (2017).
8. Sun, S. et al. The origin of high efficiency in low-temperature solution-processable bilayer organometal halide hybrid solar cells. *Energy Environ. Sci.* **7**, 399-407 (2014).
9. Yang, Y. et al. Observation of a hot-phonon bottleneck in lead-iodide perovskites. *Nat. Photon.* **10**, 53-59 (2016).
10. Miyata, A. et al. Direct measurement of the exciton binding energy and effective masses for charge carriers in organic-inorganic tri-halide perovskites. *Nat. Phys.* **11**, 582-587 (2015).
11. Saba, M. et al. Correlated electron-hole plasma in organometal perovskites. *Nat. Commun.* **5**, 5049 (2014).
12. Savenije, T. J. et al. Thermally activated exciton dissociation and recombination control the carrier dynamics in organometal halide perovskite. *J. Phys. Chem. Lett.* **5**, 2189-2194 (2014).
13. Zhang, F. et al. Brightly luminescent and color tunable colloidal CH_3_NH_3_PbX_3_ (X = Br, I, Cl) quantum dots: potential alternatives for display technology. *ACS Nano* **9**, 4533-4542 (2015).
14. Saba, M. et al. Excited state properties of hybrid perovskites. *Acc. Chem. Res.* **49**, 166-173 (2016).
15. Hu, M. et al. Distinct exciton dissociation behavior of organolead trihalide perovskite and excitonic semiconductors studied in the same system. *Small* **11**, 2164-2169 (2015).
16. Zheng, K. et al. Exciton binding energy and the nature of emissive states in organometal halide perovskites. *J. Phys. Chem. Lett.* **6**, 2969-2975 (2015).
17. Wei, Q. et al. Two-photon optical properties in individual organic-inorganic perovskite microplates. *Adv. Optical Mater.* **5**, 1700809 (2017).
18. D′Innocenzo, V. et al. Excitons versus free charges in organo-lead tri-halide perovskites. *Nat. Commun.* **5**, 3586 (2014).
19. Wu, K. et al. Temperature-dependent excitonic photoluminescence of hybrid organometal halide perovskite films. *Phys. Chem. Chem. Phys.* **16**, 22476-22481 (2014).
20. Galkowski, K. et al. Determination of the exciton binding energy and effective masses for methylammonium and formamidinium lead tri-halide perovskite semiconductors. *Energy Environ. Sci.* **9**, 962-970 (2016).
21. Jain, M. et al. Theoretical insights to excitonic effect in lead bromide perovskites. *Appl. Phys. Lett.* **118**, 192103 (2021).
22. Yang, Z. et al. Impact of the halide cage on the electronic properties of fully inorganic cesium lead halide perovskites. *ACS Energy Lett.* **2**, 1621-1627 (2017).
23. Protesescu, L. et al. Nanocrystals of cesium lead halide perovskites (CsPbX_3_, X = Cl, Br, and I): novel optoelectronic materials showing bright emission with wide color gamut. *Nano Lett.* **15**, 3692-3696 (2015).
24. Carwithen, B. P. et al. Confinement and exciton binding energy effects on hot carrier cooling in lead halide perovskite nanomaterials. *ACS Nano* **17**, 6638-6648 (2023).
25. Ruf, F. et al. Temperature-dependent studies of exciton binding energy and phase-transition suppression in (Cs, FA, MA)Pb(I, Br)_3_ perovskites. *APL Mater.* **7**, 031113 (2019).
26. Hansen, K. R. et al. Low exciton binding energies and localized exciton-polaron states in 2D tin halide perovskites. *Adv. Optical Mater.* **10**, 2102698 (2022).
27. Chen, Z. et al. Synthesis of two-dimensional perovskite by inverse temperature crystallization and studies of exciton states by two-photon excitation spectroscopy. *Adv. Funct. Mater.* **30**, 2002661 (2020).
28. Zhang, Q. et al. Excitonic properties of chemically synthesized 2D organic-inorganic hybrid perovskite nanosheets. *Adv. Mater.* **30**, 1704055 (2018).
29. Blancon, J. -C. et al. Extremely efficient internal exciton dissociation through edge states in layered 2D perovskites. *Science* **355**, 1288-1292 (2017).
30. Blancon, J. -C. et al. Scaling law for excitons in 2D perovskite quantum wells. *Nat. Commun.* **9**, 2254 (2018).
31. Cheng, B. et al. Extremely reduced dielectric confinement in two-dimensional hybrid perovskites with large polar organics. *Commun. Phys.* **1**, 80 (2018).
32. Chen, X. et al. Tuning spin-polarized lifetime in two-dimensional metal-halide perovskite through exciton binding energy. *J. Am. Chem. Soc.* **143**, 19438-19445 (2021).
33. Lu, H. et al. Highly distorted chiral two-dimensional tin iodide perovskites for spin polarized charge transport. *J. Am. Chem. Soc.* **142**, 13030-13040 (2020).
34. Zhou, N. et al. The spacer cations interplay for efficient and stable layered 2D perovskite solar cells. *Adv. Energy Mater.* **10**, 1901566 (2020).

35. Jiang, Y. et al. Reducing the impact of Auger recombination in quasi-2D perovskite light-emitting diodes. *Nat. Commun.* **12**, 336 (2021).

36. Forde, A., Tretiak, S. & Neukirch, A. J. Dielectric screening and charge-transfer in 2D lead-halide perovskites for reduced exciton binding energies. *Nano Lett.* **23**, 11586-11592 (2023).

37. Han, D. et al. Tautomeric mixture coordination enables efficient lead-free perovskite LEDs. *Nature* **622**, 493-498 (2023).

38. Chen, W. et al. Giant five-photon absorption from multidimensional core-shell halide perovskite colloidal nanocrystals. *Nat. Commun.* **8**, 15198 (2017).

39. Zhang, X. et al. Improving the five-photon absorption from core-shell perovskite nanocrystals. *J. Phys. Chem. Lett.* **14**, 7581-7590 (2023).

40. Fan, Q. et al. Near-infrared multiphoton absorption and third harmonic generation with CsPbBr_3_ quantum dots embedded in micro-particles of metal-organic frameworks. *J. Mater. Chem. C* **11**, 5788-5795 (2023).

41. Quah, H. S. et al. Multiphoton harvesting metal-organic frameworks. *Nat. Commun.* **6**, 7954 (2015).

42. Jiang, Y. et al. Frequency-upconverted stimulated emission by up to six-photon excitation from highly extended spiro fused ladder-type oligo(p-phenylene)s. *Angew. Chem. Int. Ed.* **60**, 10007-10015 (2021).

43. Guo, Z. et al. Multiphoton absorption in low-dimensional cesium copper iodide single crystals. *J. Mater. Chem. C* **8**, 16923-16929 (2020).

44. He, H. et al. Confinement of perovskite-QDs within a single MOF crystal for significantly enhanced multiphoton excited luminescence. *Adv. Mater.* **31**, 1806897 (2019).

45. Zhou, R. et al. Multiphoton excited singlet/triplet mixed self-trapped exciton emission. *Nat. Commun.* **14**, 1310 (2023).

46. Yang, D. et al. Lasing characteristics of CH_3_NH_3_PbCl_3_ single-crystal microcavities under multiphoton excitation. *Adv. Optical Mater.* **6**, 1700992 (2018).

47. Yang, D. et al. Frequency upconverted amplified spontaneous emission and lasing from inorganic perovskite under simultaneous six-photon absorption. *Opt. Lett.* **43**, 2066-2069 (2018).

48. He, G. S. et al. Multiphoton absorbing materials: molecular designs, characterizations, and applications. Chem. Rev. 108, 1245-1330 (2008).

49. Yang, D. et al. Amplified spontaneous emission from organic-inorganic hybrid iodide perovskite single crystals under direct multiphoton excitation. *Adv. Optical Mater.* **4**, 1053-1059 (2016).

50. Krishnakanth, K. N. et al. Broadband ultrafast nonlinear optical studies revealing exciting multi-photon absorption coefficients in phase pure zero-dimensional Cs_4_PbBr_6_ perovskite films. *Nanoscale* **11**, 945-954 (2019).

51. Li, J. et al. Giant two- to five-photon absorption in CsPbBr_2.7_I_0.3_ two-dimensional nanoplatelets. *Opt. Lett.* **44**, 3873-3876 (2019).

52. Syed, H. et al. Giant nonlinear optical response in triple cation halide mixed perovskite films. *Adv. Optical Mater.* **8**, 1901766 (2020).

53. Jiang, J. et al. Six-photon excited self-trapped excitons photoluminescence in lead-free halide perovskite. *Adv. Optical Mater.* **11**, 2202634 (2023).

54. Zheng, Q. et al. Frequency-upconverted stimulated emission by simultaneous five-photon absorption. *Nat. Photon.* **7**, 234-239 (2013).

55. Chon, J. W. M. et al. Three-photon excited band edge and trap emission of CdS semiconductor nanocrystals. *Appl. Phys. Lett.* **84**, 4472-4474 (2004).

56. He, J. et al. Three-photon absorption in water-soluble ZnS nanocrystals. *Appl. Phys. Lett.* **88**, 181114 (2006).

57. Wang, Y. et al. Dispersions of two-photon and three-photon absorption in GaS films from 540 to 1600 nm. *Chin. Opt. Lett.* **23**, 011901 (2025).

58. Shi, F. et al. Broadband optical nonlinearity and all-optical switching features in low-defect GaN. *Opt. Express* **31**, 32263 (2023).

59. Zhou, F. & Ji, W. Giant Three-photon absorption in monolayer MoS_2_ and its application in near-infrared photodetection. *Laser Photonics Rev.* **11**, 1700021 (2017).

60. Süsstrunk, S., Buckley, R. & Swen, S. Standard RGB color spaces. *Proc. IS&T/SID 7th Color Imaging Conference* 127-134 (1999).

61. Luo, M. R. The quality of light sources. *Color. Technol.* **127**, 75-87 (2011).

62. Luo, Z., Chen, Y. & Wu, S. -T. Wide color gamut LCD with a quantum dot backlight. *Opt. Express* **21**, 26269-26284 (2013).
